# Preparation, Characterization, and Applications of Transition Metal Dichalcogenides Nanoscrolls: Recent Development and Prospects

**DOI:** 10.3390/nano16100613

**Published:** 2026-05-16

**Authors:** Jing Ding, Xinyu Fang, Wenjie Feng, Mingxue Xu, Yang Yang, Hai Li

**Affiliations:** Key Laboratory of Flexible Electronics (KLOFE), Institute of Advanced Materials (IAM) & School of Flexible Electronics (Future Technologies), Nanjing Tech University, Nanjing 211816, China

**Keywords:** TMDC nanoscroll, spiral structure, preparation, characterization, photodetection, hydrogen evolution, optoelectronic synapse

## Abstract

Two-dimensional (2D) transition metal dichalcogenide (TMDC) nanoscrolls have attracted significant attention in recent years owing to their fascinating properties, including high specific surface area, unique electronic structure, and excellent optoelectronic performance. These properties arise from their intrinsic one-dimensional (1D) spiral scroll geometry. In this review, we systematically present the preparation methods, properties, and applications of TMDC nanoscrolls. For fabrication, we detail a variety of preparation strategies, both on substrates and in solution. Next, we discuss the characterization and physical properties of TMDC nanoscrolls. Finally, we summarize their applications in photodetection, the hydrogen evolution reaction (HER), optoelectronic synapses, and other related fields.

## 1. Introduction

Owing to their unique electronic, optical, and mechanical properties, layered transition metal dichalcogenide (TMDC) nanomaterials have attracted considerable attention in optoelectronics, energy storage, sensing, electrocatalysis, and related fields [1,2,3]. TMDCs adopt the general formula MX_2_, where M is a transition metal atom (e.g., Mo, W, Nb) and X is a chalcogen atom (S, Se, or Te). In each MX_2_ layer, an M-atom plane is sandwiched between two X-atom planes in an X-M-X stacking sequence. Adjacent layers are held together vertically by weak van der Waals interactions, allowing the exfoliation of single- or few-layer nanosheets. Taking MoS_2_ as an example, each layer consists of S-Mo-S triple planes, and neighboring layers are bonded by van der Waals forces with an interlayer spacing of 6.5 Å [4].

As two-dimensional (2D) nanomaterials, TMDC nanosheets exhibit remarkable properties, including a layer-dependent bandgap transition (indirect in bulk vs. direct in monolayers), strong light–matter interaction, significant spin–orbit coupling, valley polarization, mechanical flexibility, and catalytically active edge sites—properties that differ markedly from those of bulk materials. Nevertheless, these nanosheets face significant challenges. For instance, although the direct bandgap of TMDC nanosheets enables strong light absorption and emission, their atomic thinness limits absorption efficiency. Consequently, monolayer TMDC nanosheets absorb only 5–10% of visible light, resulting in limited photogenerated carriers and low photoresponsivity. Moreover, photocarrier trapping in atomically thin TMDC nanosheets further restricts the response speed [5].

Transforming 2D TMDC nanosheets into one-dimensional (1D) TMDC nanoscrolls could overcome these challenges. TMDC nanoscrolls not only inherit the excellent physical and chemical properties of TMDC nanosheets but also exhibit new characteristics, such as high carrier mobility, tunable band structure, enhanced optoelectronic performance, strain induced by scroll geometry, and good mechanical flexibility [6,7,8,9,10]. Compared with other 1D nanomaterials, the tubular structure of TMDC nanoscrolls endows them with distinctive electronic and optical properties [11], including a relatively wide bandgap, low dark current, high on/off ratio, good environmental stability, and enhanced carrier mobility, making them highly desirable for photodetection and sensing [12]. Furthermore, the tunable interlayer spacing and open ends of TMDC nanoscrolls offer additional advantages. The nanoscroll geometry, with its high specific surface area, effectively reduces exposure to oxygen and water vapor, thereby decreasing the degree of oxidation [13]. Owing to their one-dimensional anisotropy and high surface activity, TMDC nanoscrolls show great potential for applications in photodetection, energy storage and conversion, electrocatalysis, and synaptic devices, among others [14,15,16].

To date, various methods have been developed to prepare TMDC nanoscrolls, including organic solvent-assisted scrolling [17,18,19], centrifugal force-assisted scrolling [20], intercalation-induced scrolling [21], plasma treatment-assisted scrolling [22,23], and anisotropic stress-induced scrolling [24]. Using these methods, the length, diameter, interlayer spacing, and crystalline orientation of the resulting nanoscrolls can be controlled to a certain extent. Nevertheless, it is still highly desirable to produce high-quality nanoscrolls on a large scale with uniform structure.

In this review, we comprehensively summarize the current state of research on TMDC nanoscrolls, focusing on the diverse formation mechanisms, properties modulated by scroll geometry, and extensive applications. In the first part, we describe theoretical investigations of TMDC nanoscrolls, the developed preparation methods, and the curling mechanisms associated with each method. In the second part, we provide a detailed characterization of the physical, optical, and electrical properties of TMDC nanoscrolls. In the final part, we summarize the applications of TMDC nanoscrolls in photodetection, electrocatalysis, sensing, memory, and synaptic devices, among others.

## 2. Structure of TMDC Nanoscrolls

**Figure 1:**
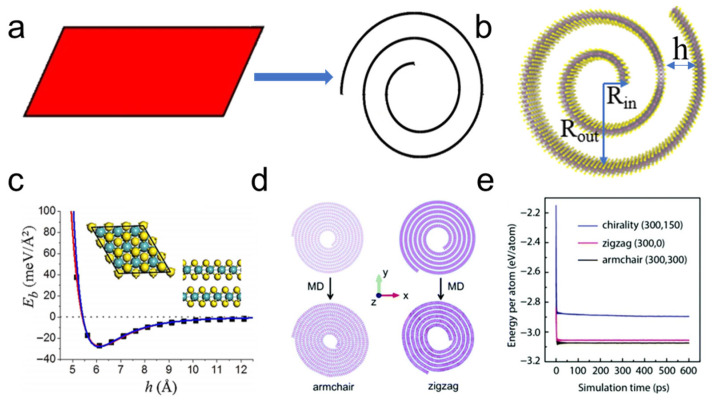
Structural illustration, energetical stability, and chirality-dependent properties of TMDC nanoscrolls. (**a**) Schematic illustration of the geometric transformation from a 2D nanosheet to a nanoscroll. Adapted with permission from Ref. [28]. Copyright 2017 IOP. (**b**) Structural diagram of a MoS_2_ nanoscroll showing the inner radius *R_in_*, outer radius *R_out_*, and interlayer spacing *h*. Adapted from Ref. [26]. (**c**) Plot of binding energy (E*_b_*) of a MoS_2_ nanoscroll as a function of interlayer spacing (*h*). Reproduced with permission from Ref. [28]. Copyright 2017 IOP. (**d**) Structural evolution of MoS_2_ sheets of the same size scrolled along the armchair (left panels) and zigzag (right panels) orientations before and after structural relaxation. (**e**) Computed energy per atom as a function of simulation time for MoS_2_ nanoscrolls with three orientations: chirality (300, 150), zigzag (300, 0), and armchair (300, 300). Reproduced with permission from Ref. [27]. Copyright 2018 RSC.

TMDC nanoscrolls refer to one-dimensional (1D) nanomaterials with a tubular structure, formed by spirally rolling up a monolayer or few-layer 2D nanosheet into an Archimedean nanoscroll (Figure 1a). TMDC nanoscrolls have open ends, and the interlayer interaction between adjacent layers is the van der Waals (vdW) force. The formation of a nanoscroll is governed by the elastic energy of the bent sheet and the interlayer interaction in the overlapping region. When the radius and number of turns of a nanoscroll are appropriate, and the interlayer interaction is sufficiently large, the nanoscroll remains more stable than its planar counterpart. The inner radius of a nanoscroll is determined thermodynamically by balancing the bending energy and the interlayer binding energy. For example, the inner radius of a MoSSe nanoscroll depends on the curvature and the interlayer binding energy [25], taking into account both the internal strain arising from structural asymmetry and the strain energy released through curling or rolling.

For a nanoscroll with inner radius *R_in_*, outer radius *R_out_*, and layer spacing *h* (Figure 1b) [26], the equilibrium configuration can be described by the following equation [27],

(1) $$\frac{2\gamma h}{D}\;=\frac{1}{R_{\mathrm{in}}}-\frac{1}{R_{\mathrm{out}}}$$

where *D* is the bending stiffness, γ is the layer binding strength, *h* is the interlayer spacing, $R_{\mathrm{in}}$ is the inner radius, and $R_{\mathrm{out}}$ is the outer radius of the nanoscroll. $R_{\mathrm{in}}$ and $R_{\mathrm{out}}$ depend strongly on the length of the scrolled nanosheet and on the interlayer spacing (*h*), provided that *h* is much smaller than the radii of the nanoscroll. Therefore, the configuration of a nanoscroll depends on the interlayer binding energy (γ) and the bending stiffness (*D*).

Liu et al. investigated the relationship between the energetically favorable interlayer spacing and the interlayer binding energy using first-principles calculations [28]. As the interlayer spacing increases, the energy per atom initially decreases and then increases. The equilibrium distance is 6.138 Å for a MoS_2_ nanoscroll (Figure 1c). Similarly, Wang et al. found that a MoS_2_ nanoscroll is in an energetically favorable state when the interlayer spacing lies in the range of 4.5–7.0 Å [27]. Crystalline orientation also plays important role in rolling up a TMDC nanosheet. Using molecular dynamics (MD) simulations, Wang et al. investigated the influence of crystalline orientation on the dominant scrolling tendency of a MoS_2_ nanosheet from a molecular-level perspective [27]. They found that the MoS_2_ nanosheet scrolled along the armchair orientation exhibited the lowest energy per atom compared with those scrolled along the zigzag or chiral orientations (Figure 1d,e). Therefore, they concluded the MoS_2_ nanosheet preferentially scrolls along the armchair direction (the Mo-S bond direction) [27].

**Figure 2:**
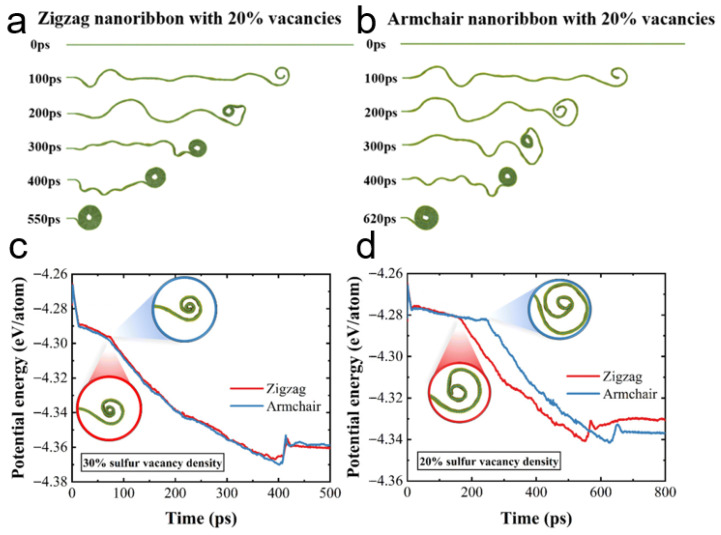
Sulfur-vacancy-induced self-curling process of (**a**) zigzag and (**b**) armchair MoS_2_ nanoribbons at a sulfur vacancy density of 20%, as revealed by MD simulations. (**c**,**d**) Potential energy per atom of zigzag and armchair MoS_2_ nanoribbons with (**c**) 30% and (**d**) 20% sulfur vacancy densities as a function of simulation time. Insets show the corresponding curling configurations at each stage. Reproduced with permission from Ref. [29]. Copyright 2023 RSC.

Yang et al. simulated the scrolling of MoS_2_ nanoribbon with varying densities of sulfur vacancies using MD simulations. A zigzag MoS_2_ nanoribbon with 20% S vacancies completely rolled into a nanoscroll after 550 ps, which was faster than the armchair MoS_2_ nanoribbon with the same vacancy density (620 ps) (Figure 2a,b). When the density of S vacancy increased to 30%, no difference in scrolling time was observed between the zigzag and armchair nanoribbons (Figure 2c,d) [29]. Using MD simulations, the same authors also investigated the preferred scrolling direction of Janus MoSSe nanosheets [25]. In contrast to MoS_2_ nanoscrolls, they found that Janus MoSSe nanoscrolls formed along the zigzag orientation exhibited lower energy than those along the armchair orientation, for both triangular and hexagonal nanosheets.

## 3. Preparation of TMDC Nanoscrolls

### 3.1. TMDC Nanoscrolls Prepared on Substrate

#### 3.1.1. Organic Solvent-Assisted Scrolling

The most common method for preparing TMDC nanoscrolls is to drop a volatile organic solvent onto monolayer TMDC nanosheets [30,31,32,33]. At room temperature, organic solvents are typically volatile liquids. During evaporation, Marangoni flows are considered to play an important role in rolling TMDC nanosheets until they form a complete nanoscroll [34].

**Figure 3:**
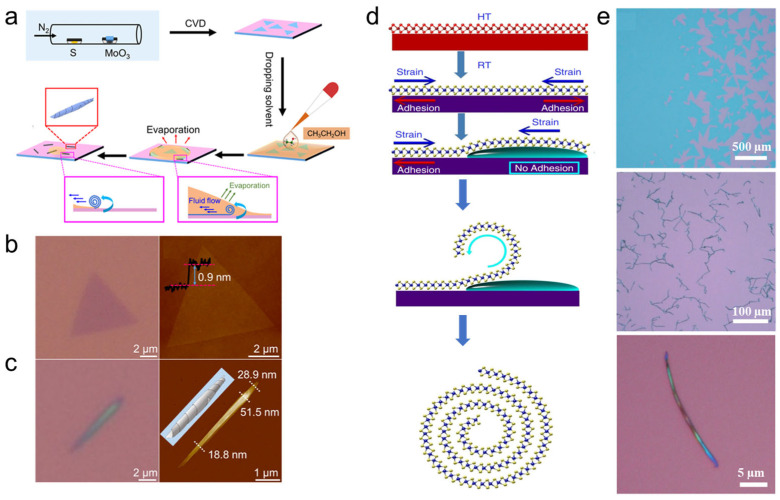
Organic solvent-assisted preparation of MoS_2_ nanoscrolls on a substrate. (**a**) Schematic of the process for preparing MoS_2_ nanoscrolls by dropping ethanol onto CVD-grown monolayer MoS_2_ nanosheets. (**b**,**c**) Optical and AFM images of (**b**) a MoS_2_ nanosheet and (**c**) a MoS_2_ nanoscroll. Reproduced with permission from Ref. [18]. Copyright 2018 ACS. (**d**) Schematic diagram of preparing MoS_2_ nanoscrolls by dropping an ethanol/water mixture onto CVD-grown monolayer MoS_2_ nanosheets. (**e**) Optical images of the MoS_2_ nanosheets and the as-prepared nanoscrolls. Reproduced from Ref. [17].

In 2018, Fang et al. prepared MoS_2_ nanoscrolls by dropping ethanol onto monolayer MoS_2_ nanosheets (Figure 3a). First, monolayer MoS_2_ nanosheets were grown directly on a SiO_2_/Si substrate via chemical vapor deposition (CVD) (Figure 3b). Then, an ethanol droplet was deposited onto the substrate to cover the CVD-grown MoS_2_ nanosheets. Due to its low surface tension and amphiphilic nature, ethanol can effectively wet the MoS_2_ and SiO_2_/Si substrate. During evaporation, a thin ethanol layer forms near the contact line, and the resulting temperature difference creates a surface tension gradient. This gradient induces fluid flow within the ethanol layer, known as Marangoni flow [34]. As the contact line recedes during evaporation, the fluid flow rolls up the edge of the MoS_2_ nanosheet until a complete MoS_2_ nanoscroll is formed (Figure 3c) [18]. Classical molecular dynamics (MD) simulations were performed to investigate the dynamic formation of MoS_2_ nanoscrolls. The results indicated that nanoscroll formation typically initiates at the edge of the MoS_2_ nanosheet [27]. The preferred scrolling direction is parallel to the armchair orientation and perpendicular to the nanosheet edge, and this direction is independent of the nanoscroll size.

An alternative method to effectively obtain MoS_2_ nanoscrolls involves immersing CVD-grown monolayer MoS_2_ nanosheets on a SiO_2_/Si substrate in ethanol solution for several hours prior to evaporation. In 2024, Qiao et al. immersed the MoS_2_ nanosheets grown on a SiO_2_/Si substrate in ethanol solution for several hours to release tensile stress, then rapidly blow-dried the sample using compressed air [35]. This process successfully yielded MoS_2_ nanoscrolls in high yield. The formation of MoS_2_-NS is partially mediated by the internal Marangoni flow within the ethanol droplet during evaporation. They noted that precise control of the scrolling process is challenging due to the hydrodynamic instability of the Marangoni flow, which often results in distorted or excessively scrolled structures. By blowing away the ethanol droplet in a timely manner, the scrolling process can be halted at an appropriate intermediate stage, yielding partially scrolled MoS_2_ nanoscrolls with excellent axial uniformity.

In 2018, Zheng et al. successfully prepared MoS_2_, WS_2_, MoSe_2_ and WSe_2_ NSs by dropping a mixture of ethanol and water with a volume ratio of 2:1 onto CVD-grown monolayer TMDC nanosheets [17]. The preparation process is illustrated in Figure 3d. First, monolayer TMDC nanosheets were obtained on a SiO_2_/Si substrate by CVD at a high temperature (≥720 °C). Because of the difference in thermal expansion coefficients between the TMDC nanosheets and the SiO_2_/Si substrate (Figure 3d), strain was generated in the nanosheets upon cooling to room temperature. After the aqueous ethanol solution was dropped onto the TMDC nanosheets, the liquid film intercalated the interface between the nanosheets and the substrate. Consequently, the edges of the TMDC nanosheets were released from the substrate. Thus, the adhesion force between the edges and the substrate was reduced, releasing the built-in strain and curling the nanosheet edges into scrolls.

**Figure 4:**
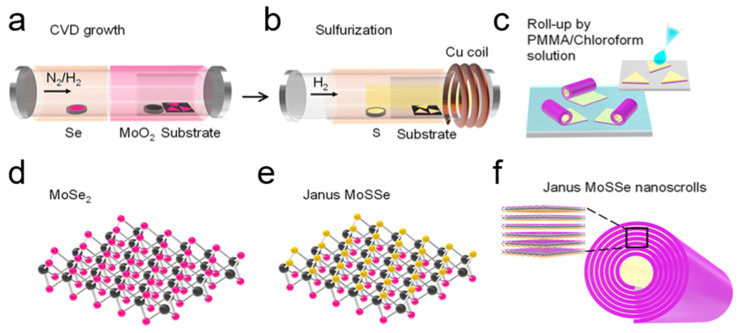
Organic-solvent-assisted preparation of Janus MoSSe nanoscrolls. (**a**) CVD growth of a monolayer MoSe_2_ nanosheet. (**b**). Replacement of the top Se atoms by S via hydrogen plasma treatment to form Janus MoSSe nanosheets. (**c**). Rolling up Janus MoSSe nanosheets by spin-coating a PMMA/chloroform solution. (**d**,**e**) Schematic structures of (**d**) MoSe_2_ and (**e**) MoSSe nanosheets. (**f**) Structural diagram of a Janus MoSSe nanoscroll. Reproduced with permission from Ref. [36]. Copyright 2024 ACS.

In addition to ethanol, other volatile organic solvents, such as acetone [31], isopropanol [33] and chloroform solutions [36], are also used to assist the formation of TMDC nanoscrolls. In 2022, Ghosh et al. prepared WS_2_/MoS_2_ heterojunction nanoscrolls by dropping acetone onto a WS_2_/MoS_2_ heterojunction film [32]. In 2024, Kaneda et al. reported the fabrication of MoSSe NSs by spin-coating a PMMA/chloroform solution onto Janus MoSSe monolayer nanosheets (Figure 4) [36]. First, monolayer MoSe_2_ (or WSe_2_) nanosheets were grown on a SiO_2_/Si substrate by CVD (Figure 4a,b) [36]. At room temperature, the Se atoms on the top surface of the monolayer were replaced by S atoms using hydrogen plasma treatment (Figure 4c), forming a Janus MoSSe monolayer (Figure 4e) [37]. After that, cracks were formed because of the tensile strain introduced into grains. A drop of polymethyl methacrylate (PMMA)/chloroform solution was then spin-coated onto the substrate, and chloroform molecules permeated into the interface between the nanosheets and the SiO_2_/Si substrate owing to their low surface tension, releasing the edges of the Janus MoSSe nanosheets from the substrate (Figure 4c). Thereafter, the edges of local strain-induced cracks and grain boundaries in Janus nanosheets served as nucleation sites, triggering the directional spontaneous scrolling to form Janus MoSSe NSs (Figure 4f). Yang et al. investigated the spontaneous formation process of Janus TMDC NSs using molecular dynamics simulations [38]. It was found that the relaxation of intrinsic strain was the driving force that initiated the scrolling of a MoSSe nanoribbon. When the Se atoms at one end of the MoSe_2_ nanosheet are partially replaced by S atoms, the out-of-plane atomic asymmetry induces internal stress relaxation, leading to spontaneous curling. Moreover, the bending stiffness, spontaneous curling curvature, interlayer distance, interlayer interaction, and length of the MoSSe nanoribbon can significantly influence the inner radius of MoSSe NSs [38]. Furthermore, armchair and zigzag Janus MoSSe nanoribbons exhibit similar scrolling behavior, indicating that the scrolling process is insensitive to the crystalline orientation.

By dropping volatile organic solvents onto TMDC nanosheets, high-quality TMDC nanoscrolls with large dimensions can be produced in large quantities within a short time. However, residual organic solvents encapsulated within the scroll and the loosely scrolled structure remain challenges for enhancing the performance of TMDC nanoscrolls. These residues are found to act as charge trapping centers, which are responsible for the giant hysteresis and memory effect [35]. Meanwhile, the solvent induced scattering and charge impurity effects decreased carrier mobility and introduced device instability [39].

#### 3.1.2. Alkali Solvent-Assisted Scrolling

To release TMDC nanosheets from the underlying substrate, overcoming the adhesion force between them is the key factor in successfully preparing TMDC nanoscrolls. Volatile organic solvents are typically used to scroll monolayer TMDC nanosheets. However, the interface between the TMDC nanosheets and the substrate cannot be effectively intercalated by volatile organic solvents when the adhesion force is too strong. Moreover, organic solvents are ineffective at curling thick TMDC nanosheets with high bending stiffness. It has been reported that Marangoni flow within an evaporating organic droplet cannot effectively roll up the edges of bilayer or thick TMDC nanosheets to form scrolls [12,40], owing to either the increased bending stiffness of the nanosheet or its strong adhesion to the substrate. Therefore, alternative methods that overcome the strong adhesion force or high rigidity of TMDC nanosheets are desirable for preparing nanoscrolls.

**Figure 5:**
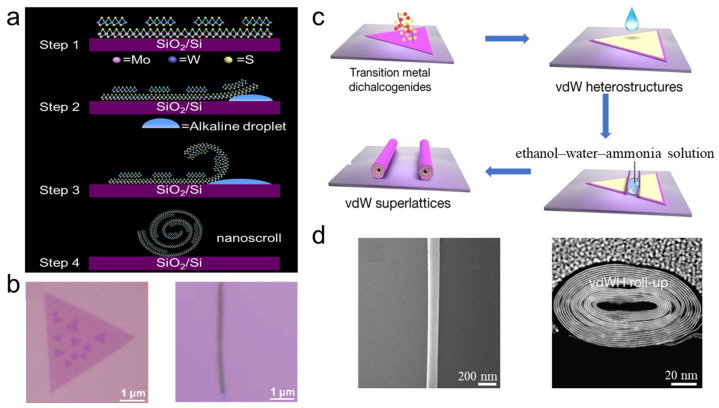
Etching of the SiO_2_ substrate with alkaline solution to roll up TMDC nanosheets. (**a**) Schematic illustration of the preparation of WS_2_/MoS_2_ nanoscrolls using an alkaline solution. (**b**) Optical images of a WS_2_/MoS_2_ nanosheet and nanoscroll before and after dropping the alkaline solution. Reproduced with permission from Ref. [12]. Copyright 2020 Springer Nature. (**c**) Schematic diagram of the preparation of van der Waals (vdW) heterostructure nanoscrolls using an ethanol-water-ammonia mixture. (**d**) SEM and cross-sectional STEM images of the as-prepared vdW heterostructure nanoscrolls. Reproduced with permission from Ref. [40]. Copyright 2021 Springer Nature.

In 2020, Wang et al. prepared TMDC heterostructure nanoscrolls by dropping alkaline solution onto hetero-bilayer TMDC nanosheets grown on a SiO_2_/Si substrate [12]. First, large-area bilayer WS_2_/MoS_2_ heterostructures were grown on a SiO_2_/Si substrate by CVD (Figure 5a). Subsequently, 50 μL of 0.1 M KOH or NaHCO_3_ solution was dropped onto the bilayer WS_2_/MoS_2_ heterostructures. With increasing reaction time, the alkaline solution etched the top layer of the SiO_2_ film and then penetrated into the interface between the hetero-bilayer and the underlying substrate. As a result, the adhesion force between the hetero-bilayer and the SiO_2_/Si substrate was eliminated. Subsequently, the released hetero-bilayer WS_2_/MoS_2_ nanosheets spontaneously rolled up from the edges and formed nanoscrolls (Figure 5b).

To transform bilayer or thick TMDC heterostructures into 1D nanoscrolls, Zhao et al. used a mixed ethanol-water-ammonia solution to delaminate the thick heterostructures from the underlying substrate [40]. First, a monolayer TMDC nanosheet was grown on a SiO_2_/Si substrate by a modified CVD process with controllable reverse flow. The as-grown TMDC nanosheet was used as a template for epitaxially growing another 2D nanosheet on top, yielding vertically stacked van der Waals (vdW) hetero-bilayer nanosheets (Figure 5c,d), such as SnS_2_/WSe_2_ and NbSe_2_/MoSe_2_. A drop of the ethanol-water-ammonia mixture was then added onto the TMDC hetero-bilayer. After the SiO_2_/Si substrate was etched by the alkaline solution, the SnS_2_/WSe_2_ heterostructure was peeled off from the substrate and spontaneously rolled up to form nanoscrolls [40]. Various 2D/2D vdW heterostructures, including MoSe_2_/WSe_2_, SnS_2_/MoS_2_, MoS_2_/WS_2_, SnSe_2_/WSe_2_, Cr_5_Te_8_/WSe_2_, and In_2_Se_3_/WSe_2_, have been successfully scrolled using this method. In addition, thin films and 1D nanowires can also be encapsulated into TMDC nanoscrolls, extending the capability of this method for creating mixed-dimensional vdW heterostructure nanoscrolls.

Zero-dimensional (0D) nanoparticles can also be encapsulated into TMDC nanoscrolls using alkaline solution. After PbI_2_ nanoparticles were deposited on monolayer MoS_2_ nanosheets, they were immersed in a mixture of ammonia and isopropanol to prepare the PbI_2_/MoS_2_ nanoscrolls [41]. Similarly, Zhang et al. prepared BaTiO_3_/MoS_2_ nanoscrolls by dropping NaHCO_3_ solution onto BaTiO_3_ nanoparticles-decorated monolayer MoS_2_ nanosheets [42]. Overall, the use of alkaline solution to overcome the adhesion force between TMDC nanosheets and the substrate has advanced the fabrication of TMDC nanoscrolls. It is a highly reproducible process for preparing heterojunction nanoscrolls with high yield and large dimensions. However, etching of the underlying substrate and residual solvent trapped within the nanoscrolls remain challenges.

#### 3.1.3. Dragging Water Droplet on Hot Substrate

**Figure 6:**
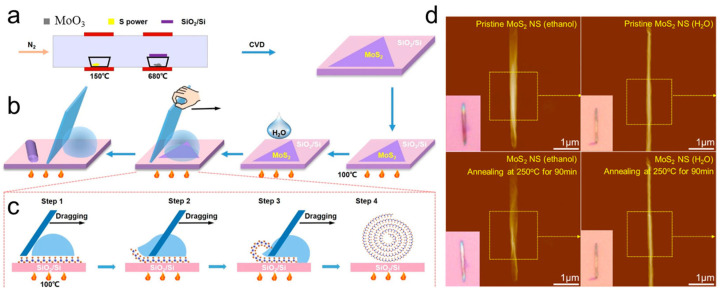
Formation of MoS_2_ nanoscrolls by dragging a water droplet on CVD-grown MoS_2_ nanosheets at 100 °C. (**a**) Schematic illustration of the CVD process for MoS_2_ nanosheet growth on a SiO_2_/Si substrate. (**b**) Schematic of the water-assisted scrolling process of MoS_2_ nanosheets at 100 °C. (**c**) Stepwise illustration of the formation of a MoS_2_ nanoscroll by dragging a water droplet (Steps 1–4) at 100 °C on a SiO_2_/Si substrate. (**d**) AFM images of pristine and annealed MoS_2_ NSs prepared by dropping ethanol and by dragging a water droplet, respectively. Reproduced with permission from Ref. [39]. Copyright 2022 ACS.

Although organic solvents and alkaline solutions have been successfully used to prepare TMDC nanoscrolls, the device performance of these nanoscrolls is inevitably degraded by residual solvent trapped within them. In 2022, Zhao et al. reported a solvent-free method to fabricate clean, tightly packed TMDC nanoscrolls by dragging a water droplet across monolayer TMDC nanosheets on a hotplate (Figure 6). First, large-area monolayer MoS_2_ nanosheets were grown by CVD and then heated on a hotplate at 100 °C. Subsequently, a deionized (DI) water droplet was deposited on the hot MoS_2_ nanosheets and dragged from one end to the other at a speed of 3 mm/s (Figure 6a). The formation of a nanoscroll is illustrated in Figure 6b. At 100 °C, the adhesion force between the MoS_2_ nanosheet and the underlying SiO_2_/Si substrate was weaker than that at room temperature. As the water droplet moved, the edge of the MoS_2_ nanosheet curled first, and then a nanoscroll formed spontaneously (Figure 6c). As shown in Figure 6d, the height of the MoS_2_ NS prepared using ethanol (referred to as MoS_2_-EtOH NS) decreased by approximately one-third after heating at 250 °C for 30 min, which was attributed to the evaporation of ethanol molecules encapsulated within the MoS_2_ NS. In contrast, the height of the MoS_2_ NS (H_2_O) remained almost unchanged under the same conditions. Because the MoS_2_ nanosheet is hydrophobic, water molecules could not be encapsulated within the nanoscroll, resulting in a solvent-free MoS_2_ NS. This method can produce clean, tightly packed TMDC nanoscrolls with high yield. However, it is not applicable to materials that are sensitive to water or heat [39].

#### 3.1.4. Spin-Coating-Assisted Scrolling

**Figure 7:**
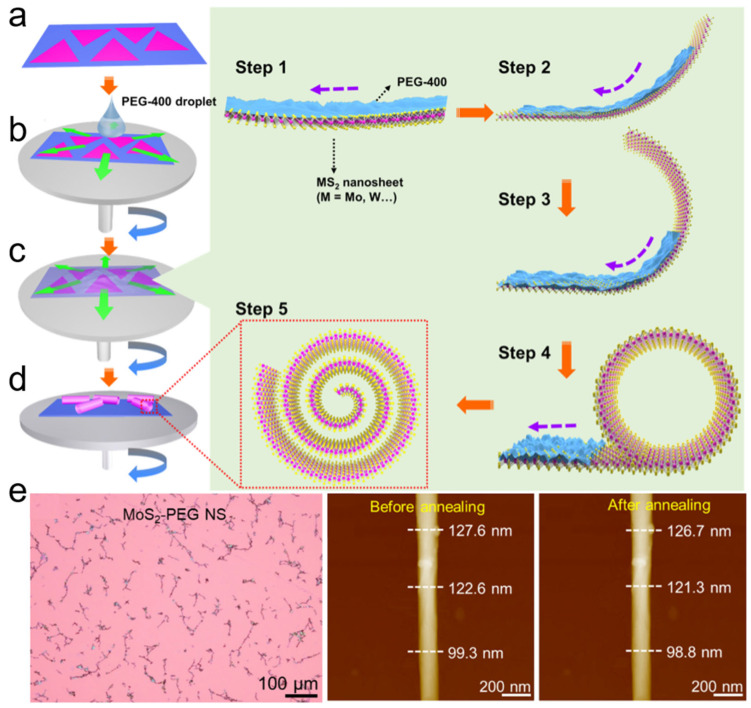
Fabrication of TMDC nanoscrolls by spin-coating PEG-400 on monolayer TMDC nanosheets. (**a**) Monolayer TMDC nanosheets grown by CVD. (**b**) Continuous dropping of PEG-400 onto CVD-grown TMDC nanosheets at 3000 rpm for 30 s. (**c**) A PEG droplet spreads onto the nanosheets and flows to the substrate edge under centrifugal force. (**d**) As-prepared TMDC nanoscrolls obtained by spin-coating PEG-400. (**e**) Optical and AFM height images of MoS_2_ nanoscrolls. Reproduced with permission from Ref. [20]. Copyright 2024 ACS.

Spin-coating is a widely used technique for uniformly distributing liquid onto a substrate that is rotated at a controlled speed. When a liquid droplet is deposited onto the rotating substrate, it spreads outward and flows from the center to the edge under centrifugal force. In this process, the liquid flow can overcome the adhesion force between the nanosheet and the substrate, potentially forming a nanoscroll. In 2024, Yu et al. prepared TMDC nanoscrolls by spin-coating a viscous polyethylene glycol (PEG) droplet onto CVD-grown monolayer TMDC nanosheets [20], as shown in Figure 7. First, monolayer TMDC nanosheets were grown on a SiO_2_/Si substrate by CVD (Figure 7a). Then, viscous PEG droplets were spin-coated continuously onto the nanosheets at 3000 rpm (Figure 7b). As the PEG flowed outward from the center to the edge of the substrate (Figure 7c), the edge of TMDC nanosheet curled up and nanoscroll formed spontaneously (Figure 7d).

PEG appears as a viscous liquid at room temperature when its molecular weight is less than 800 g/mol, i.e., PEG-800. In the experiment, PEG-400 with a viscosity of 37–45 mm^2^/s was spin-coated onto monolayer TMDC nanosheets. Compared with other low-viscosity solvents (such as deionized water and ethanol), PEG-400 flows at an appropriate speed under centrifugal force. During spin-coating, the interaction between the flowing PEG and the TMDC nanosheets is stronger than the adhesion between the nanosheets and the substrate, which drives the edges of the nanosheets to roll up into a scrolled structure. Unlike volatile organic solvents and alkaline solutions, PEG molecules can be effectively removed by soaking in deionized water at 100 °C. After annealing at 250 °C, the height of the MoS_2_-PEG NS remained almost unchanged (Figure 7e), indicating the PEG molecules are not trapped inside the nanoscroll. The MoS_2_-PEG NS exhibited significantly higher carrier mobility, photosensitivity, photoresponsivity, and external quantum efficiency (EQE) than those of the MoS_2_ nanosheet and the MoS_2_-EtOH NS [43]. The enhanced performance can be attributed to confined carrier motion along the one-dimensional scrolled structure, the increased light-absorption cross-section of the nanoscroll, and the absence of oxygen and water within the tightly packed scroll structure. TMDC nanoscrolls prepared by this method offer the advantages of high yield, a compact and clean structure, and an environmentally friendly process.

#### 3.1.5. Plasma Treatment-Assisted Scrolling

**Figure 8:**
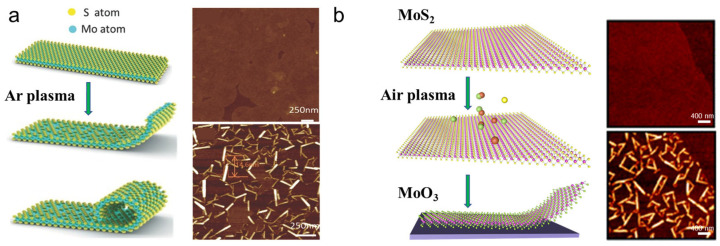
Preparation of TMDC nanoscrolls by plasma treatment of TMDC nanosheets. (**a**) Ar plasma-assisted preparation of MoS_2_ nanoscrolls. Adapted with permission from Ref. [22]. Copyright 2016 Wiley. (**b**) Air plasma-assisted fabrication of MoO_3_ nanoscrolls. Adapted with permission from Ref. [23]. Copyright 2017 RSC.

In 2016, Zhang et al. prepared MoS_2_ nanoscrolls by treating CVD-grown monolayer MoS_2_ nanosheets with argon plasma at 150 °C [22]. Under plasma bombardment, sulfur atoms were directly removed from the top surface of the MoS_2_ nanosheet when the kinetic energy of the plasma overcame the Mo-S binding energy. As a result, sulfur vacancies initially appeared at defects and reactive grain boundaries. Consequently, the MoS_2_ lattice was disrupted, and out-of-plane strain was generated due to the sulfur vacancies. This stress in the MoS_2_ basal plane curled the edge up and drove the formation of nanoscrolls along defective sites and grain boundaries, as shown in Figure 8a. As the plasma power increased, the time required to form MoS_2_ nanoscrolls decreased. Conversely, longer time was required to trigger scrolling of MoS_2_ nanosheets under low-power plasma. Kinks formed when the adjacent edges of the MoS_2_ nanosheet were not parallel. MD simulations confirmed that sulfur vacancies induced atomic asymmetry and provided the driving force for scrolling. As the sulfur vacancy density increased from 15% to 30%, the MoS_2_ nanoribbon exhibited an accelerated scrolling rate, and less time was required to form the nanoscroll [29].

By replacing argon with air, Wang et al. obtained MoO_3_ and WO_3_ nanoscrolls by treating MoS_2_, WS_2_, MoSe_2_, and WSe_2_ nanosheets with plasma. Monolayer TMDC nanosheets were first prepared using mechanical exfoliation or CVD and then placed into a plasma cleaner at a chamber pressure of 40–80 mTorr. MoO_3_ and WO_3_ nanoscrolls were obtained by air plasma treatment at 18 W for 2–3 s, as shown in Figure 8b. When MoS_2_ nanosheets were treated by air plasma, sulfur atoms in the top surface were replaced by oxygen atoms, forming Mo-O bonds. Consequently, lattice distortion occurred, introducing strain and rolling up the edge of the MoO_3_ nanosheet to form a nanoscroll [23].

The preparation of TMDC nanoscrolls by plasma treatment is convenient and solvent-free. Nevertheless, several challenges remain. First, the size of the resulting TMDC nanoscrolls is too small for practical applications. The length of TMDC nanoscrolls prepared by this method is typically several hundred nanometers. Second, the original nanosheets are damaged by plasma bombardment, and the resulting nanoscrolls are amorphous with poor crystalline quality. Third, the plasma treatment method is ineffective for multilayer 2D materials. This limitation arises from the strong interlayer interaction between the oxidized layer and the underlying TMDC layer. Consequently, the induced stress is insufficient to overcome the adhesion force and drive the formation of a nanoscroll.

#### 3.1.6. Rapidly Quenching Induced Scrolling

**Figure 9:**
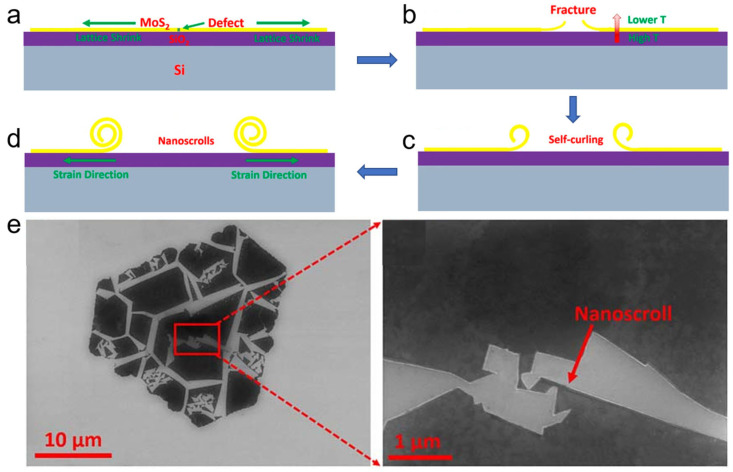
Preparation of MoS_2_ nanoscrolls on a SiO_2_/Si substrate by rapid quenching after CVD growth. (**a**) CVD growth of MoS_2_ nanosheets on a SiO_2_/Si substrate. (**b**) Under the strain induced by quenching, a S vacancy acts as a crack nucleation site. (**c**) The newly formed crack edge curls up to minimize surface energy. (**d**) A MoS_2_ nanoscroll forms spontaneously. (**e**) Large-scale and magnified SEM images of MoS_2_ nanoscrolls. Adapted with permission from Ref. [44]. Copyright 2016 Wiley.

Hao et al. prepared MoS_2_ nanoscrolls by introducing strain into CVD-grown monolayer MoS_2_ nanosheet via rapid quenching [44], as shown in Figure 9. Monolayer MoS_2_ nanosheets were first grown on a SiO_2_/Si substrate by CVD (Figure 9a) and then rapidly quenched to room temperature at a cooling rate of approximately 300 °C/min. Owing to the difference in thermal expansion coefficients, a lattice contraction mismatch arose between the MoS_2_ nanosheet and the SiO_2_/Si substrate during quenching. Because the MoS_2_ nanosheet cooled faster than the SiO_2_/Si substrate, it shrank more rapidly as the temperature quickly decreased to room temperature (Figure 9b). During CVD growth, sulfur vacancies inevitably formed on the MoS_2_ nanosheets, which acted as crack nucleation sites owing to the strain induced by rapid quenching. Consequently, the MoS_2_ nanosheet cracked and curled up at these defective sites. To minimize the surface free energy, the curled edges spontaneously rolled up until a MoS_2_ nanoscroll formed (Figure 9c,d). Cracks typically propagated along the zigzag direction, resulting in fracture along an energetically favorable orientation (Figure 9e).

It has been reported that the structural transition from a nanosheet to a nanoscroll depends on the competition between van der Waals (vdW) interactions and elastic bending energy. In this regard, the vdW interactions in the overlapping regions of the MoS_2_ nanosheet reduce the surface free energy by an amount that exceeds the increase in elastic bending energy caused by scrolling. Therefore, the scrolling process occurs spontaneously to form a MoS_2_ nanoscroll. However, the low yield and incomplete curling of TMDC nanosheets achieved with this method hinder its practical application.

This section describes six methods for fabricating TMDC nanoscrolls on a substrate: organic solvent-assisted scrolling, alkali solvent-assisted scrolling, dragging a water droplet on a hot substrate, spin-coating-assisted scrolling, plasma treatment-assisted scrolling, and rapid quenching-induced scrolling.

The key step in rolling up TMDC nanosheets from a substrate is overcoming the adhesion force between the nanosheet and the substrate. In organic solvent-assisted scrolling, Marangoni flow generated during solvent evaporation rolls up the edges of TMDC nanosheets. Alkaline solutions completely eliminate adhesion by etching the SiO_2_ substrate. Dragging a water droplet or spin-coating a polyethylene glycol (PEG) droplet across a TMDC nanosheet uses liquid flow to roll up the nanosheet edges, producing nanoscrolls free of solvent residues. Plasma treatment introduces surface defects and induces strain due to lattice distortion, which rolls up the nanosheet edges to form nanoscrolls. During rapid quenching, the difference in thermal expansion coefficients between the TMDC nanosheet and the substrate generates strain, leading to crack formation and subsequent scrolling.

Nevertheless, these preparation methods still face several challenges. First, the yield of nanoscrolls is too low for practical applications. Second, solvent residues may become encapsulated inside the nanoscrolls, although they can be partially removed by annealing. Third, achieving uniform nanoscrolls with high controllability remains difficult. Considerable efforts are therefore required to address these challenges.

### 3.2. TMDC Nanoscrolls Prepared in Solution

The aforementioned methods for preparing TMDC nanoscrolls are conducted on substrates. Nevertheless, the scalable synthesis of TMDC nanoscrolls with controlled dimensions (diameter, length, and number of layers) on substrates remains a significant challenge, yet it is crucial for practical applications. Therefore, it is highly desirable to develop a promising method for preparing TMDC nanoscrolls in large quantities in a controlled manner. Exploring the formation of TMDC nanoscrolls in solution could provide an alternative approach to addressing this challenge.

#### 3.2.1. Shear Force Assisted Scrolling

**Figure 10:**
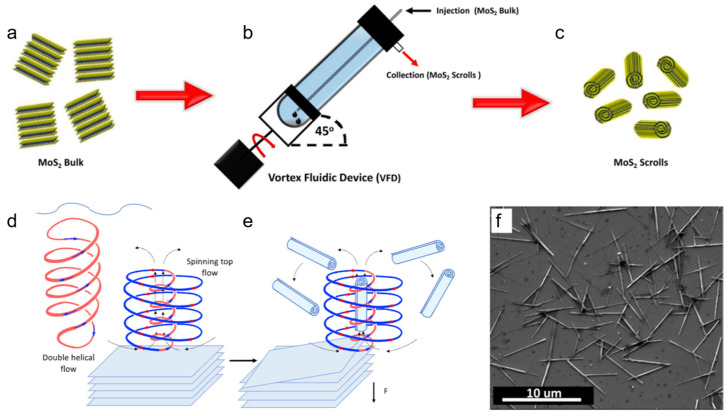
Shear-force-assisted preparation of MoS_2_ nanoscrolls in a vortex fluidic device (VFD). (**a**–**c**) Schematic diagrams of the experimental process for fabricating MoS_2_ nanoscrolls in a VFD. (**d**,**e**) The two topological fluid flows governing shear stress: (**d**) the typhoon-like spinning top (ST) (at 4000 rpm) and (**e**) the double-helix (DH) flow (at 8000 rpm). (**f**) SEM image of MoS_2_ scrolls fabricated in the VFD under continuous flow. Reproduced with permission from Ref. [45]. Copyright 2022 ACS.

Alharbi et al. transformed 2D MoS_2_ nanosheets into MoS_2_ nanoscrolls under continuous flow in a homemade vortex fluidic device (VFD), as shown in Figure 10. First, MoS_2_ powders with a lateral size of approximately 1.5 µm was dispersed in a mixture of ethanol, water, and DMF in a volume ratio of 1:1:1 and ultrasonicated for 30 min (Figure 10a). The mixture was then placed into the VFD and processed at a rotation speed of 4000 rpm, a flow rate of 0.45 mL/min, and a tilt angle of 45° (Figure 10b). Finally, the product was collected and vacuum-dried to obtain the nanoscrolls (Figure 10c) [45].

Within the VFD, mechanical energy was converted into high shear stress through topological fluid flows at the micrometer scale, including typhoon-like spinning top (ST) flow from the tube bottom and double-helix (DH) flow from the thin film. At a low rotation speed of 4000 rpm, the shear stress was dominated primarily by the typhoon-like ST flow (Figure 10d). At a high rotation speed of 8000 rpm, the DH flow arising from twisted Faraday wave vortices dominated the shear stress. The ST flow generated shear stress on the MoS_2_ surface and induced exfoliation to produce MoS_2_ nanosheets. The as-exfoliated nanosheets were then curled into nanoscrolls with the aid of a chiral upward flow (Figure 10e), located at the center of the ST [46,47]. SEM and TEM characterization revealed that the as-prepared nanoscrolls had a diameter of less than 0.2 μm and a length ranging from 3 to 10 μm (Figure 10f).

Compared with existing preparation methods for TMDC nanoscrolls, the VFD method offers the following advantages. On one hand, the yield of nanoscrolls can be increased by extending the processing time. On the other hand, the exfoliation and scrolling of MoS_2_ nanosheets can be achieved simultaneously under optimized conditions.

#### 3.2.2. Sonication Assisted Scrolling

**Figure 11:**
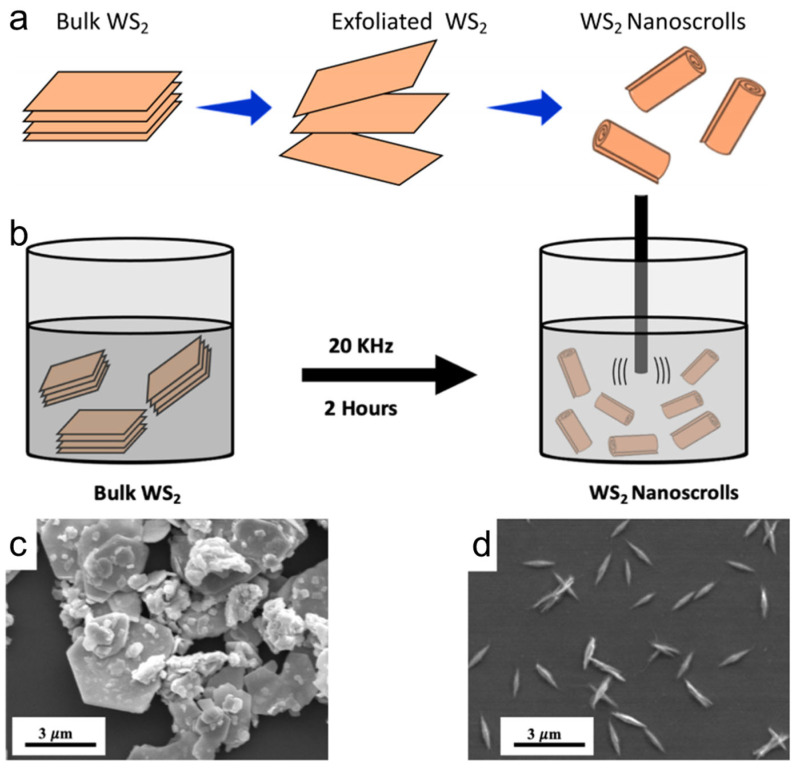
(**a**,**b**) Preparation of WS_2_ nanoscrolls by ultrasonication of WS_2_ powder in DMF (5 mg/mL) at low frequency. SEM images of (**c**) bulk WS_2_ and (**d**) the as-prepared WS_2_ nanoscrolls. Reproduced from Ref. [48].

Thaar M.D. Alharbi also fabricated WS_2_ nanoscrolls by sonicating WS_2_ powder in DMF. In the experiment, a low-frequency (20 kHz) probe ultrasonication was used, as high-frequency ultrasonication could damage the crystal structure of the WS_2_ powder. WS_2_ powder was first dispersed in DMF to form a colloid suspension using a bath sonicator for 15 min. The resulting solution was then placed in a probe sonicator for 2 h to prepare WS_2_ nanoscrolls. During the first hour of sonication, WS_2_ platelets with a lateral size of approximately 2 μm were first exfoliated into nanosheets by shock stress waves induced by cavitation bubble collapse. The as-exfoliated WS_2_ nanosheets were then transformed into 1D WS_2_ nanoscrolls by sonicating for an additional hour. In this process, continuous cavitation bubble collapse enabled the WS_2_ nanosheets to overcome surface energy, resulting in scrolling of the exfoliated nanosheets (Figure 11). During sonication, the temperature was maintained below 30 °C [48].

This method is simple, rapid, and low-cost, achieving a yield of WS_2_ nanoscrolls of up to 90%. Moreover, it can directly prepare WS_2_ nanoscrolls from bulk WS_2_ without the use of surfactants or pretreatment steps. However, the length of the resulting WS_2_ nanoscrolls is less than 1 μm, which limits their practical application.

#### 3.2.3. Supercritical Fluid Assisted Scrolling

When the temperature and pressure of a fluid are above the critical point, the fluid is in a supercritical state. A supercritical fluid (SCF) exhibits both gas-like and liquid-like physicochemical properties, including a high diffusion coefficient, near-zero interfacial tension, pressure-tunable solvating power, and low viscosity [49,50]. Owing to these intriguing properties, SCFs have been applied to the liquid exfoliation of layered materials, as they are expected to efficiently penetrate the gaps between adjacent layers and delaminate them.

**Figure 12:**
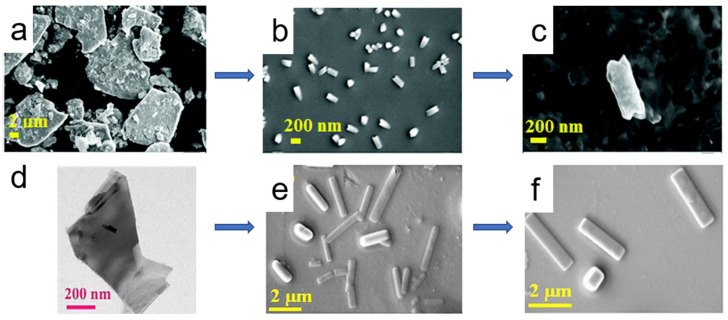
Preparation of TMDC nanoscrolls by treating TMDC powder in supercritical fluid. (**a**–**c**) FE-SEM images of (**a**) bulk MoS_2_ and (**b**,**c**) MoS_2_ nanoscrolls. Adapted with permission from Ref. [51]. Copyright 2016 RSC. (**d**–**f**) FE-SEM images of (**d**) WS_2_ nanosheet and (**e**,**f**) WS_2_ nanoscrolls. Adapted with permission from Ref. [52]. Copyright 2020 Elsevier.

By treating MoS_2_ nanosheets with SCF, Pitchai Thangasamy and Marappan Sathish successfully fabricated MoS_2_ and WS_2_ nanoscrolls in DMF solution within 30 min (Figure 12) [51]. MoS_2_ powder was added to DMF and ultrasonicated for 5 min to form a suspension. The suspension was then poured into a sealed stainless-steel reactor and heated in a furnace at 400 °C for 30 min. The reactor was then removed and immediately quenched in an ice-water bath. The supernatant was centrifuged to collect the nanoscrolls. During SCF processing, the bulk MoS_2_ was first delaminated into MoS_2_ nanosheets. To minimize the surface energy, the as-exfoliated MoS_2_ nanosheets spontaneously rolled up to form 1D nanoscrolls. SEM characterization indicated that the as-prepared nanoscrolls had a diameter of 50–150 nm and a length of 0.2–3 μm (Figure 12a–c). In 2022, they also prepared the one-dimensional WS_2_ nanoscrolls from bulk WS_2_ powder using the same one-pot SCF processing for 30 min [52] (Figure 12d–f). The high-temperature SCF processing provided the energy for interlayer separation, exfoliating the bulk WS_2_ into few-layer nanosheets (3–10 layers). Owing to their high surface energy, the as-exfoliated 2D WS_2_ nanosheets were unstable and spontaneously formed a 1D spirally scrolled structure through a thermodynamically driven process. Partially curled WS_2_ nanosheets were observed after SCF processing for 15 min, confirming the intermediate state of transformation.

This method enables the mass production of TMDC nanoscrolls. The crystallinity and catalytic activity of the TMDC nanosheets are well preserved, making them suitable for optoelectronics and energy storage. However, the length of the resulting nanoscrolls is relatively short.

#### 3.2.4. Self-Assembling of Amphiphilic Materials-Assisted Scrolling

**Figure 13:**
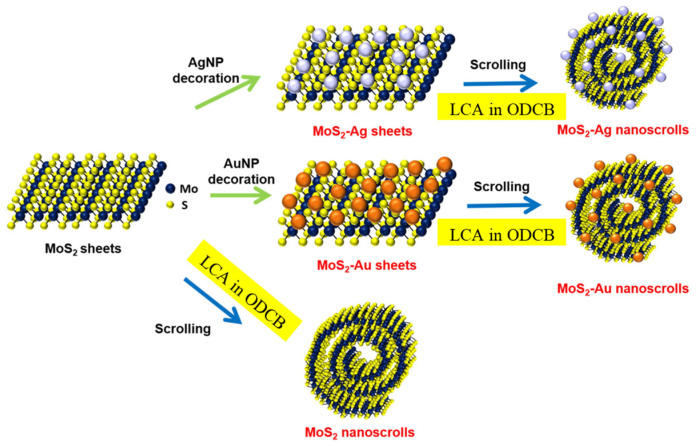
Schematic illustration of the preparation of MoS_2_ nanoscrolls, as well as MoS_2_@Ag and MoS_2_@Au nanoscrolls, via the self-assembly of LCA. Adapted with permission from Ref. [56]. Copyright 2017 IOP.

In 2017, Hwang et al. prepared MoS_2_ nanoscrolls in solution through the self-assembly of an amphiphilic material [53], N-(2-aminoethyl)-3α-hydroxy-5β-cholan-24-amide (LCA), as shown in Figure 13. First, LCA was dissolved in o-dichlorobenzene (ODCB) and heated to 60 °C. MoS_2_ nanosheets were exfoliated by ultrasonication. The LCA solution was then poured into the MoS_2_ solution and stored at room temperature for 24 h to obtain MoS_2_ nanoscrolls. Using this method, TMDC nanoscrolls such as MoS_2_, MoSe_2_, and MoTe_2_ nanoscrolls, have been successfully prepared [54].

The formation of MoS_2_ nanoscrolls by this method can be explained as follows. LCA molecules contain amine functional groups, which exhibit strong binding affinity for MoS_2_ nanosheets, particularly at the edges. One side of the MoS_2_ edge may have a slightly stronger interaction with LCA molecules, leading to a subtle interaction difference between the two sides. As a result, the edge of the MoS_2_ nanosheet bends, and the adsorbed LCA molecules gradually self-assemble into a fiber. After the bent MoS_2_ edge completes the first scroll turn, the LCA molecules continue to self-assemble. Consequently, the MoS_2_ nanosheet transforms into a nanoscroll encapsulating a self-assembled LCA fiber.

In addition to bare MoS_2_ nanosheet, nanoparticles-decorated MoS_2_ nanosheets can also be transformed into nanoscrolls by adding LCA in solution. When LCA molecules were added to a solution of Pt nanoparticle-decorated MoS_2_ nanosheets, MoS_2_@Pt nanoscrolls were obtained (Figure 13) [55]. Similarly, MoS_2_@Au and MoS_2_@Ag nanoscrolls were also prepared by adding LCA (Figure 13) [56]. By adjusting the concentration of LCA and the type of nanoparticles, the diameter and size of the resulting nanoscrolls can be controlled. Although TMDC nanoscrolls can be produced in large quantities by this method, the use of small-sized TMDC nanosheets leads to short nanoscrolls.

#### 3.2.5. Pulsed Laser Ablation (PLA) Assisted Scrolling

Owing to hydrogen bonding at room temperature, a mixture of choline chloride and urea in a 1:2 molar ratio remains liquid, forming a deep eutectic solvent (DES). Using DES as a confinement medium, Betancourt et al. prepared MoSe_2_ nanoscrolls via pulsed laser ablation (PLA) [57]. Owing to the high-oxygen environment, MoO_x_ nanoparticles are preferentially formed by PLA in water, inhibiting the formation of large-sized nanosheets. The MoSe_2_ nanosheets prepared in DES are larger than those prepared in water by PLA, which is attributed to the lower polarity of DES compared with that of water. In addition, DES has low oxygen content, and the as-prepared MoSe_2_ nanosheets have a highly crystalline structure, demonstrating the advantages of DES for PLA. In DES, the MoSe_2_ nanosheets tend to be flexible and undergo deformation at the edges owing to the strong hydrogen bonding interactions of the environment. The edge of a MoSe_2_ nanosheet can be bent by the interlayer van der Waals forces acting vertically on the MoSe_2_ surface, which are thought to overcome the hydrogen bonding interactions of DES, thereby inducing scrolling. The DES used in this method is a low-vapor-pressure and recyclable solvent, making it more environmentally friendly than volatile organic solvents. The size distribution of nanoscrolls can be optimized by adjusting the processing parameters.

#### 3.2.6. Stirring Magnetically Assisted in Solution

**Figure 14:**
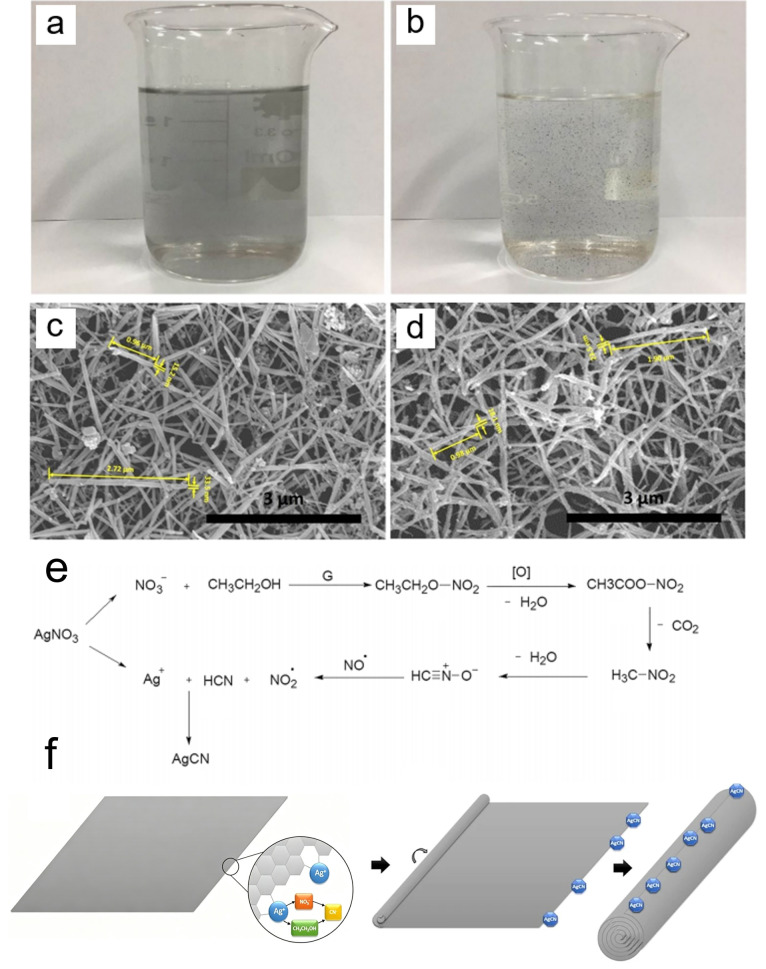
Preparation of TMDC nanoscrolls by magnetic stirring of AgNO_3_ and TMDC nanosheets in ethanol. (**a**,**b**) Digital photographs of (**a**) TMDC nanosheets and (**b**) TMDC nanoscrolls dispersed in ethanol. (**c**,**d**) SEM images of (**c**) MoS_2_ and (**d**) WS_2_ nanoscrolls. Reproduced from Ref. [58]. (**e**) Schematic illustration of AgCN formation. Reproduced from Ref. [60]. (**f**) Schematic of the preparation of a nanoscroll driven by AgCN nanoparticles adsorbed at the edges of a nanosheet. Reproduced from Ref. [58].

In 2018, Wang et al. used a magnetic stirring method to fabricate graphene, h-BN, and TMDC nanoscrolls with high yield [58]. Two-dimensional nanosheets with a thickness of 1–5 layers were exfoliated using a supercritical CO_2_ method in a shear mixer [59]. The as-prepared nanosheets and AgNO_3_ were mixed and magnetically stirred in ethanol for 30 min at room temperature. The nanosheet dispersion changed from a uniform gray solution to a clear solution with suspended black precipitates (Figure 14a,b), which were confirmed to be nanoscrolls by SEM and TEM characterization (Figure 14c,d).

When 2D nanosheets are mixed with AgNO_3_ in ethanol solution, the active dangling bonds at the nanosheet edges trigger the formation of cyanide ion (CN^−^) through cleavage of the C-C bond in ethanol and the N-O bond in the nitrate ion [60]. Silver cyanide (AgCN) nanoparticles were formed in situ when Ag^+^ ions met CN^−^ ions (Figure 14e), which were observed at the edges of the nanoscrolls. The as-generated AgCN nanoparticles modified the surface electron density of the nanosheets, which affected the adsorption state of ethanol on the nanosheets and increased their surface energy. As the amount of AgCN nanoparticles increased, the surface energy of the nanosheets gradually accumulated. Once the energy exceeded a critical threshold, the nanosheets curled from the edges to reduce their surface energy (Figure 14f). During curling, the edges of the nanosheets gradually overlapped with the inner layers. Thus, interlayer van der Waals forces became the driving force for continued curling until the nanoscroll was fully formed. Therefore, the formation of AgCN nanoparticles at the edges of the nanosheets played a significant role in driving the scrolling process.

This section describes five methods for preparing TMDC nanoscrolls in solution, which serve as a distinct complement to substrate-based preparation techniques. Bulk TMDCs are first exfoliated into thin layers, followed by spontaneous scrolling driven by shear flow in a vortex fluidic device (VFD), impact stress during ultrasonication, elevated temperature and pressure above the critical point of supercritical fluids, and edge-localized particles induced by magnetic stirring. This process is fundamentally different from in situ scrolling on substrates.

These solution-based preparation methods have both advantages and challenges. The VFD and ultrasonication methods achieve yields exceeding 90% within several hours, whereas the supercritical fluid method completes the process in 30 min, making it suitable for mass production. The self-assembly method enables the facile preparation of composite nanoscrolls (e.g., MoS_2_@Au) through co-assembly of LCA molecules and nanoparticles (Pt, Au, Ag), thereby expanding their functionality. Supercritical fluids and pulsed laser ablation (PLA) in deep eutectic solvent media preserve the lattice integrity of TMDCs well, whereas intense ultrasonication or high shear forces may induce edge defects. However, most nanoscrolls prepared by solution-based methods have lengths ranging from 1 to 10 μm, which is much shorter than the hundred-micrometer-scale nanoscrolls obtained on substrates. This size difference arises because the initial dimensions of bulk TMDC powders in solution are typically below 10 μm.

In summary, TMDC nanoscrolls prepared on substrates are suitable for device integration and fundamental research, whereas those prepared in solution are used in fields requiring large quantities of nanoscrolls, such as energy storage, catalysis, and composite functional materials.

In this section, we have discussed the preparation of TMDC nanoscrolls to date. Despite the apparent diversity of the methods described in Section 3, three prerequisites are necessary for successful scrolling of TMDC nanosheets: (i) an external stimulus must overcome the adhesion force on the substrate or the bending energy in solution to initiate curling; (ii) the interlayer interaction between adjacent layers must be strong enough to maintain the scrolled structure on the substrate or in solution; and (iii) the nanosheet must have sufficiently low bending stiffness. Thus, although the specific initiation mechanisms vary, the fundamental physics underlying scroll formation is unified: any process that locally detaches a flexible, strained nanosheet leads to spontaneous scrolling that minimizes surface free energy. The advantages, challenges, and underlying principles of each method are summarized in Table 1. The preparation methods are also classified according to the types of nanoscrolls produced in Table 2.

## 4. Properties of TMDC Nanoscrolls

In this section, we summarize the property investigation of TMDC nanoscrolls using techniques such as transmission electron microscopy (TEM), second harmonic generation (SHG), Raman spectroscopy, photoluminescence (PL) spectroscopy, circular dichroism spectroscopy, photoconductive atomic force microscopy (AFM), X-ray photoelectron spectroscopy (XPS), and semiconductor characterization systems. These results reveal significant differences between TMDC nanosheets and nanoscrolls in terms of structure, crystalline defects, electrical and optoelectronic performance, and magnetoresistance.

### 4.1. Morphology

**Figure 15:**
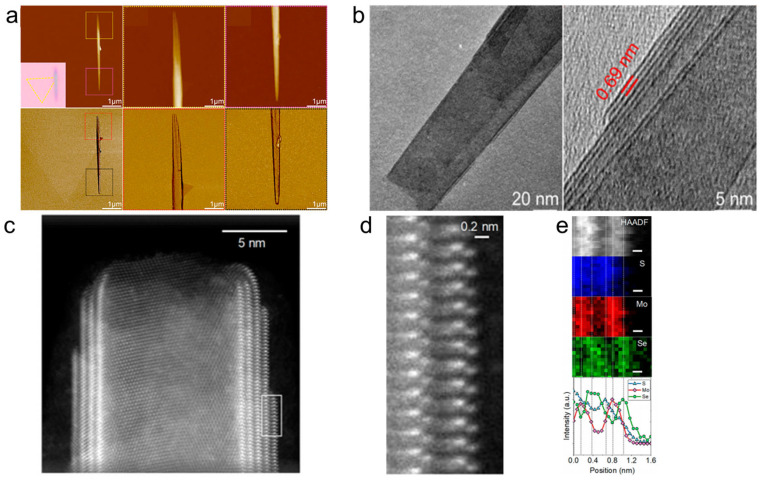
Morphological characteristics of TMDC nanoscrolls. (**a**) AFM image of a MoS_2_ nanoscroll transformed from a triangular MoS_2_ nanosheet. Reproduced with permission from Ref. [39]. Copyright 2022 ACS. (**b**) TEM images of a WS_2_ nanoscroll. Reproduced with permission from Ref. [18]. Copyright 2018 ACS. (**c**) HAADF-STEM image of a Janus MoSSe nanoscroll, showing the layer spacing. (**d**) Magnified STEM image showing the three-atom-thick structure of an individual layer. (**e**) HAADF-STEM image and EELS maps of a Janus MoSSe nanoscroll. Reproduced with permission from Ref. [36]. Copyright 2024 ACS.

Atomic force microscopy (AFM) can clearly resolve the morphology of nanoscrolls, including length, diameter, and spiral structure. The AFM image clearly shows that the MoS_2_ nanoscroll is transformed in situ from a triangular nanosheet (Figure 15a). The magnified AFM phase image clearly shows that the MoS_2_ nanoscroll has an Archimedean screw structure. Transmission electron microscopy (TEM) is used to observe the high-resolution structure of TMDC nanoscrolls, particularly their crystalline structure and interlayer spacing. As shown in Figure 15b, the TEM image of a WS_2_ nanoscroll shows a closely packed layered structure with an interlayer spacing of 0.69 nm. This value is consistent with the measured thickness of a single-layer nanosheet. Figure 15c shows the multiwall structure of a Janus MoSSe nanoscroll observed by high-angle annular dark-field scanning transmission electron microscopy (HAADF-STEM) [19,21]. The magnified STEM image clearly shows the three-atom-thick structure of an individual layer (Figure 15d). The HAADF-STEM image and electron energy loss spectroscopy (EELS) maps of the Janus nanoscroll are shown in Figure 15e. As observed from the spatial distribution in the EELS maps, the inner and outer sides of the Mo atoms are occupied by S and Se atoms, respectively, confirming the replacement of surface S atoms by Se atoms.

### 4.2. Optical Properties

#### 4.2.1. Second Harmonic Generation

**Figure 16:**
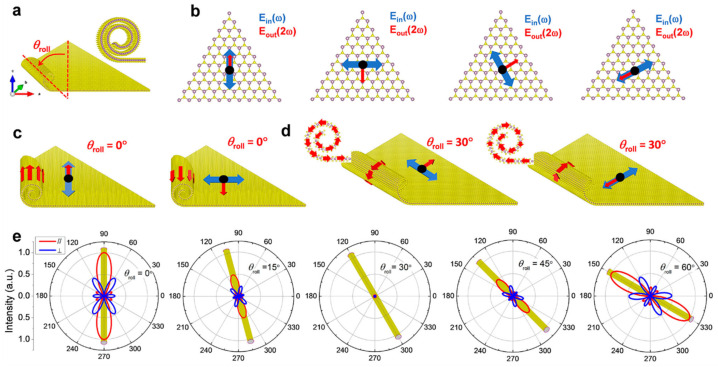
Second harmonic generation (SHG) of MoS_2_ nanoscroll. (**a**) Schematic structure of a MoS_2_ nanoscroll. (**b**) Electric fields (or dipoles) of the second harmonic (SH) induced by a polarized incident laser at different orientations. (**c**,**d**) Schematics of SH electric fields (dipoles) in MoS_2_ nanoscrolls with (**c**) *θ*_roll_ = 0° and (**d**) *θ*_roll_ = 30°, induced by a polarized incident laser oriented along and perpendicular to the nanoscroll axis, respectively. (**e**) Calculated polarization-resolved SHG emission patterns of MoS_2_ nanoscrolls with different chiralities. Reproduced with permission from Ref. [64]. Copyright 2020 ACS.

As a second-order nonlinear optical process, second harmonic generation (SHG) is widely used in applications such as optical frequency conversion, interface spectroscopy, and ultrashort pulse characterization [62]. Odd-numbered 2H-MoS_2_ layers exhibit inversion symmetry breaking and show observable SHG responses, whereas even-numbered layers do not generate SHG owing to the presence of inversion symmetry [63]. In 2020, Qian et al. found that MoS_2_ nanoscrolls exhibited strong chirality-dependent SHG owing to their reduced symmetry (Figure 16) [64]. Unlike previous polarization-resolved SHG measurements that relied on sample rotation, they collected the SHG signal by rotating the polarization of the incident laser, thereby improving accuracy and consistency. Interestingly, the MoS_2_ nanoscroll exhibited an SHG signal two orders of magnitude higher than that of a monolayer MoS_2_ nanosheet, a phenomenon that was sensitive to the chirality of the nanoscroll and arose from the superposition of the SH electric fields from the nanoscroll walls. The angle between the nanoscroll axis and the armchair orientation of the triangular MoS_2_ nanosheet was defined as the rolling direction, denoted as *θ*_roll_ (Figure 16a). The laser polarization was set parallel and perpendicular to the nanoscroll axis, respectively. The induced SH electric field was oriented parallel or perpendicular to the polarized incident laser along the armchair or zigzag direction, respectively (Figure 16b). When *θ*_roll_ = 0°, the SH electric fields from the nanoscroll walls were aligned with the nanoscroll axis when the incident laser was polarized along the axis direction (Figure 16c). In this case, the SHG efficiency was greatly enhanced because the total SH dipole experienced no orientation loss. When the incident laser was polarized perpendicular to the nanoscroll axis, the overall SHG signal was weakened owing to the out-of-plane component of the incident electric field on the vertical sidewall of the MoS_2_ nanoscroll. When the MoS_2_ nanosheet was rolled up along the zigzag orientation (*θ*_roll_ = 30°), the SH dipole was oriented spirally around the nanoscroll axis when the incident laser was polarized both along and perpendicular to the axis (Figure 16d). Therefore, the total SHG electric fields counteract each other, resulting in a very weak SHG signal (Figure 16e).

**Figure 17:**
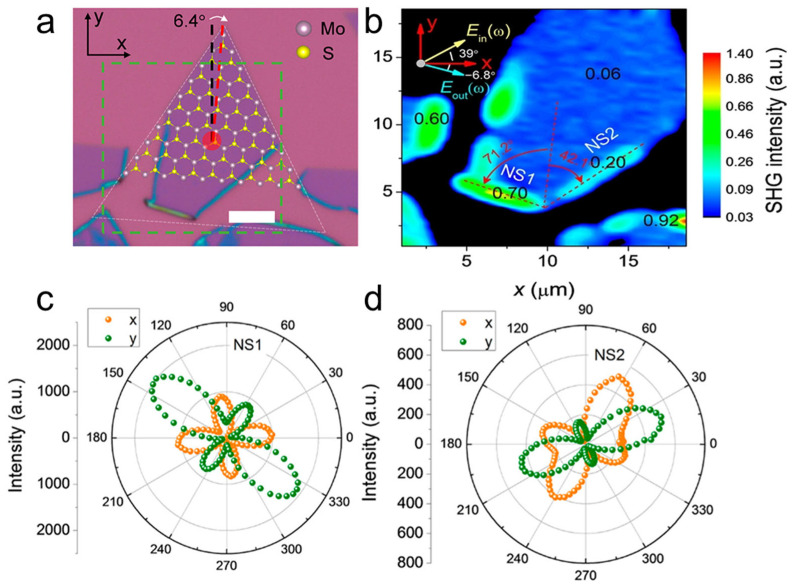
(**a**) Optical image of a CVD-grown monolayer MoS_2_ nanosheet with partially rolled nanoscrolls. (**b**) SHG intensity mapping of the monolayer MoS_2_ nanosheet and the nanoscrolls within the green dashed box in (**a**). (**c**,**d**) Polarization-resolved SHG patterns of (**c**) NS1 and (**d**) NS2 MoS_2_ nanoscrolls measured experimentally with the SH electric field along the x and y directions, respectively. Reproduced with permission from Ref. [64]. Copyright 2020 ACS.

Figure 17a,b shows the optical image and polarization-resolved SHG mapping of a MoS_2_ nanosheet and a MoS_2_ nanoscroll, respectively. The monolayer MoS_2_ nanosheet exhibited a uniform but weak SHG signal with an intensity of 0.06. In contrast, the MoS_2_ nanoscrolls with *θ*_roll_ = 71.2° (NS1) and *θ*_roll_ = 42.1° (NS2) exhibited strong SHG signals, which were 12 and 3.3 times higher than that of the monolayer MoS_2_ nanosheet, respectively. Figure 17c,d shows the polarization-resolved SHG emission patterns of NS1 and NS2 measured experimentally, where the SH electric field was polarized along the x and y directions, respectively. Unlike the isotropic four-fold petal pattern observed in the monolayer MoS_2_ nanosheet, anisotropic four-petal patterns with enhanced intensities were observed for NS1 and NS2, demonstrating the symmetry breaking induced by scrolling [62,65,66,67]. The highest total SHG intensities (I_x_ + I_y_) of NS1 and NS2 were 95 and 34 times higher than those of the monolayer MoS_2_ nanosheet, respectively, when the incident laser was polarized nearly along the nanoscroll axis.

#### 4.2.2. Nonlinear Circular Dichroism (SHG-CD)

**Figure 18:**
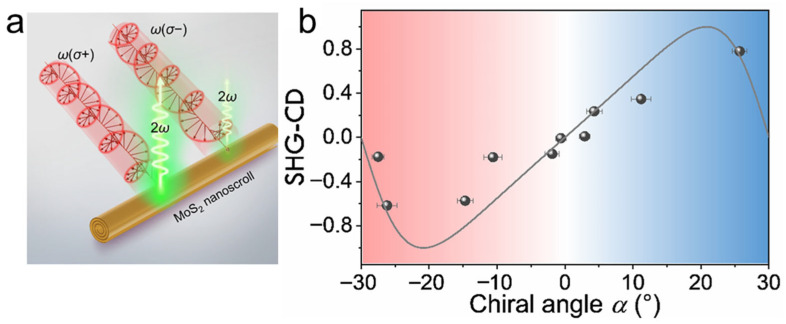
(**a**) Schematic diagram of the emitted SHG (2ω) from MoS_2_ nanoscrolls excited by left-circularly polarized light ω (σ^+^) and right-circularly polarized light ω (σ^−^). (**b**) Degree of SHG-CD signal as a function of chiral angle, α. Reproduced with permission from Ref. [68]. Copyright 2025 ACS.

Nonlinear circular dichroism (CD) spectroscopy is used to characterize the nonlinear optical responses of chiral materials excited by the left-circularly polarized (LCP) light (σ^+^) and right-circularly polarized (RCP) light (σ^−^) (Figure 18a), respectively. By replacing the half-wave plate with a quarter-wave plate (QWP), Xue et al. investigated nonlinear CD in chiral MoS_2_ nanoscrolls by measuring circular polarization-dependent SHG [68]. Monolayer MoS_2_ nanosheets were first grown by CVD and then self-assembled into chiral nanoscrolls in a mixture of isopropanol and water. The chiral MoS_2_ nanoscrolls exhibited distinct nonlinear responses under LCP and RCP excitation, generating SHG-CD signals. The degree of structural asymmetry was described by the chiral angle, α, defined as the angle between the armchair orientation of the MoS_2_ lattice and the nanoscroll axis. α was positive when the nanoscroll axis was oriented counterclockwise relative to the armchair direction of the MoS_2_ lattice, and negative when oriented in the opposite direction. The strength of the SHG-CD signal was defined as (*I*_σ+_ − *I*_σ−_)/(*I*_σ+_ + *I*_σ−_). By tuning α in the range from −30° to 30°, the strength of the SHG-CD signal can be continuously modulated from −1 to 1 (Figure 18b). When α = 0° or ±30°, no CD signal was observed because the nanoscroll possessed inversion symmetry. When α = ±24°, the CD signal reached a maximum of 0.8, indicating the strongest CD effect under these conditions. The ability to manipulate the chirality of MoS_2_ nanoscrolls provides a new route for precisely controlling the nonlinear optical response.

#### 4.2.3. Raman Spectroscopy

Raman spectroscopy is a fast, non-destructive technique for characterizing the electronic and crystalline structure of 2D nanomaterials. Raman peaks of 2D nanomaterials provide rich information on frequency, line shape, intensity, and full width at half maximum, which are related to lattice orientation, number of layers, defects, doping, stacking order, strain, and other factors.

**Figure 19:**
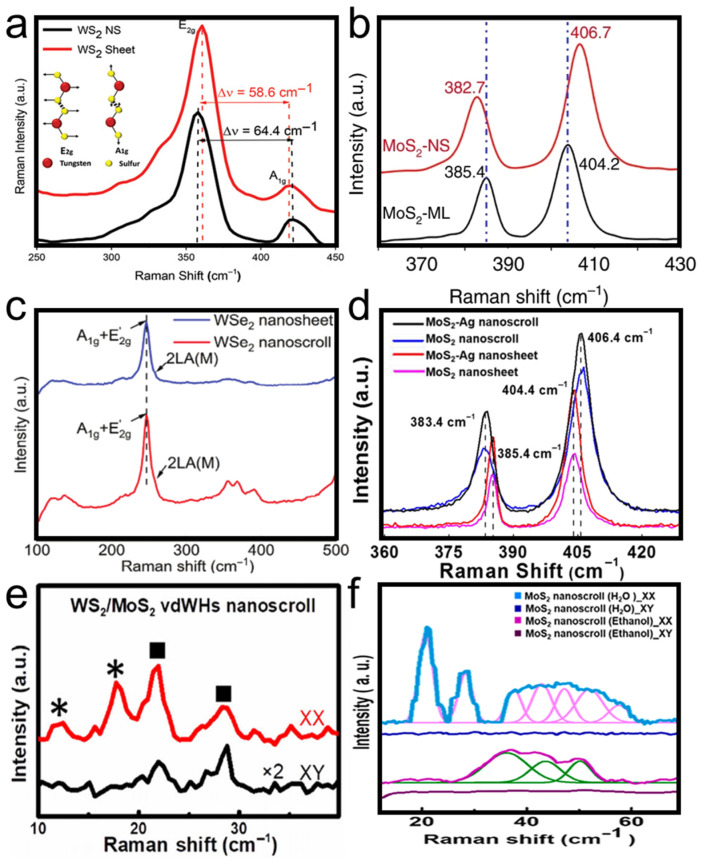
Raman spectra of (**a**) WS_2_ nanosheet and nanoscroll, and (**b**) MoS_2_ nanosheet and nanoscroll. Reproduced with permission from Ref. [69]. Copyright 2020 Wiley. Reproduced from Ref. [17]. (**c**) Raman spectra of a bilayer WSe_2_ nanosheet and a WSe_2_ nanoscroll. Reproduced with permission from Ref. [19] Copyright 2024 Wiley. (**d**) Raman spectra of a MoS_2_ nanosheet, a MoS_2_ nanoscroll, a MoS_2_-Ag nanosheet, and a MoS_2_-Ag nanoscroll. Reproduced with permission from Ref. [74]. Copyright 2021 ACS. (**e**) Ultralow-frequency (ULF) Raman spectra of a WS_2_/MoS_2_ nanoscroll. The peaks marked by asterisks and square symbols are LB mode and shear mode peaks, respectively. Reproduced with permission from Ref. [12]. Copyright 2020 Springer Nature. (**f**) ULF Raman spectra of MoS_2_ nanoscrolls prepared by dropping an ethanol solution and by dragging a water droplet on a hot plate, respectively. Reproduced with permission from Ref. [39]. Copyright 2022 ACS.

Raman spectroscopy also plays an important role in characterizing TMDC nanoscrolls [69]. Figure 19a shows the Raman spectra of a WS_2_ nanosheet and a WS_2_ nanoscroll [69]. The A_1g_ mode of the WS_2_ nanoscroll exhibits a blue shift due to increased interlayer interaction [70,71]. In contrast, the E_2g_ mode of the WS_2_ nanoscroll exhibits a red shift, which is attributed to increased uniaxial strain on the basal plane caused by bending of the nanoscroll. Furthermore, the frequency difference between the E_2g_ and A_1g_ modes of the WS_2_ nanoscroll is larger than that of the WS_2_ nanosheet, indicating an increase in long-range Coulomb interactions owing to structural and stacking deformation [72,73]. Similar phenomena are also observed in the Raman spectra of MoS_2_ nanoscrolls [17], WSe_2_ nanoscrolls [19], and MoS_2_-Ag nanoscrolls (Figure 19b–d) [74].

High-frequency Raman spectroscopy (>100 cm^−1^) is primarily used to probe intralayer vibrations in 2D nanomaterials. Although ultralow-frequency (ULF) in-plane and out-of-plane interlayer vibrations (<100 cm^−1^) are relative weak and close to the Rayleigh line, they are sensitive to vdW interactions and coupling in stacked 2D nanosheets. Therefore, ULF Raman spectroscopy is often used to accurately characterize the number of layers and stacking order. The main ULF vibration modes include the TA mode (transverse acoustic mode), shear mode, and LB mode (layer breathing mode). As shown in Figure 19e, the ULF Raman spectrum of WS_2_/MoS_2_ nanoscrolls shows four peaks located at 12.4, 17.7, 21.9, and 28.2 cm^−1^, respectively, under *XX* polarization. The disappearance of the two peaks marked with asterisks under *XY* polarization indicates that they are LB mode peaks, confirming the existence of interfacial coupling between adjacent layers in the WS_2_/MoS_2_ nanoscrolls. The other two peaks, marked with square symbols (21.9 and 28.2 cm^−1^), are still observed under *XY* polarization, implying that they are shear mode peaks [12].

Figure 19f shows the ULF Raman spectra of MoS_2_ nanoscrolls under *XX* and *XY* polarizations. The MoS_2_ nanoscrolls were prepared by dropping ethanol solution and by dragging a water droplet on a hot plate, respectively, and are referred to as MoS_2_ NS-W and MoS_2_ NS-E. After peak deconvolution, MoS_2_ NS-E exhibited three Raman peaks under *XX* polarization. Under the same conditions, MoS_2_ NS-W showed seven Raman peaks. All of these Raman peaks disappeared under *XY* polarization, indicating that they were LB mode peaks and confirming out-of-plane interlayer interactions in the MoS_2_ nanoscroll. Compared with MoS_2_ NS-E, MoS_2_ NS-W exhibited more ULF LB mode peaks, implying that MoS_2_ NS-W had stronger interlayer interaction between adjacent layers than MoS_2_ NS-E did. This demonstrates that the scroll structure of MoS_2_ NS-W is more compact than that of MoS_2_ NS-E [39].

#### 4.2.4. Photoluminescence (PL) Spectroscopy

**Figure 20:**
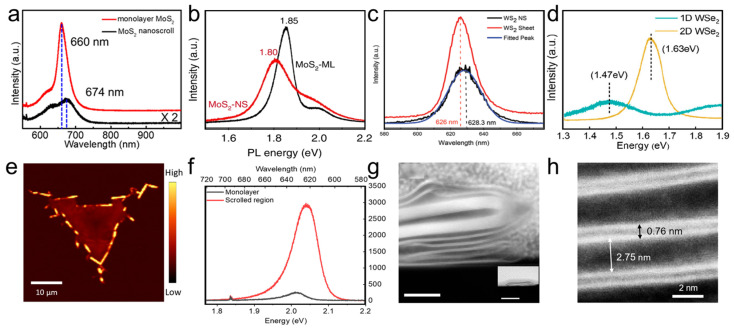
(**a**) PL spectra of a monolayer MoS_2_ nanosheet and a MoS_2_ nanoscroll. Reproduced with permission from Ref. [18]. Copyright 2018 ACS. (**b**) PL spectra of a MoS_2_ monolayer and a MoS_2_ nanoscroll. Reproduced from Ref. [17]. (**c**,**d**) PL spectra of (**c**) a WS_2_ nanoscroll and nanosheet and (**d**) a WSe_2_ nanoscroll and nanosheet. Reproduced with permission from Ref. [69]. Copyright 2020, Wiley. Reproduced with permission from Ref. [77]. Copyright 2024 ACS. (**e**) PL mapping and (**f**) PL spectra of a triangular MoS_2_ flake with a partially scrolled structure. (**g**) TEM and (**h**) HRTEM images of a loosely assembled MoS_2_ nanoscroll. Reproduced with permission from Ref. [31]. Copyright 2022 ACS.

Monolayer TMDC nanosheets are known to exhibit strong photoluminescence (PL) owing to their direct bandgaps [75]. However, the PL intensity of TMDC nanosheets decreases with increasing thickness owing to the direct-to-indirect bandgap transition. The bandgap of a TMDC nanosheet can be tuned by introducing local strain [76]. In a TMDC-NS, the monolayer TMDC nanosheet is rolled up into a curved structure. Consequently, strain is inevitably distributed in the basal plane because of the bent geometry. Furthermore, a TMDC-NS behaves as a multilayer structure to some extent. Therefore, the PL of a TMDC-NS is influenced by its scrolled structure. As shown in Figure 20, the PL peaks of various TMDC nanoscrolls exhibited a clear red shift compared with those of monolayer TMDC nanosheets [17,18,31,77], indicating a change in the electronic structure of the nanoscroll. The red shift in the PL peaks may arise from stacking effects and deformation-induced strain in the nanoscroll. However, the PL peak of the MoS_2_ nanoscroll with a loosely assembled structure showed a blue shift compared with that of the nanosheet (Figure 20a,b) [31]. Moreover, the loosely scrolled MoS_2_ nanoscroll exhibited a much stronger PL peak than the monolayer MoS_2_ nanosheet (Figure 20e,f) [31]. This enhanced PL peak was attributed to the considerably large interlayer spacing of 2.75 nm in the MoS_2_ nanoscroll (Figure 20g,h), which resulted from the intercalation of acetone molecules during rapid scrolling. Similar blue-shifted PL peaks were also observed in a WSe_2_ nanoscroll prepared from a bilayer nanosheet and in a MoS_2_–Ag nanoscroll (Figure 20d) [19,74,78], suggesting that these nanoscrolls may also possess a loosely scrolled or inhomogeneously folded structure.

**Figure 21:**
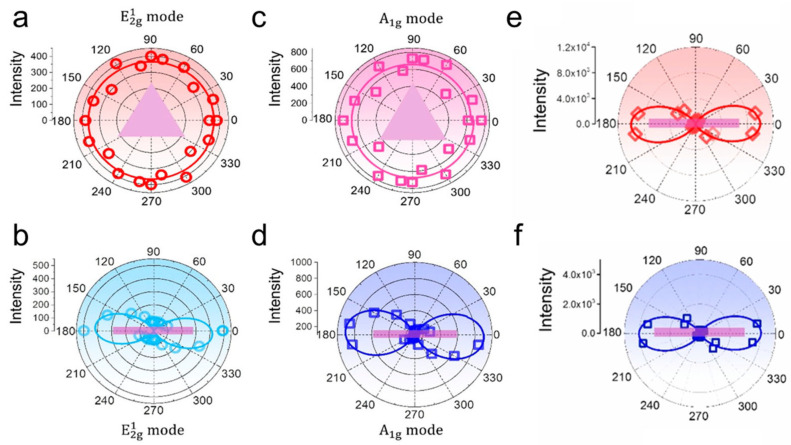
(**a**,**c**) Polar plots of the Raman intensities of the (**a**) $E_{2g}^{1}$ and (**c**) A_1g_ modes for a MoS_2_ nanosheet. (**b**,**d**) Polar plots of the Raman intensities of the (**b**) $E_{2g}^{1}$ and (**d**) A_1g_ modes for a MoS_2_ nanoscroll. (**e**,**f**) Polar plots of the A and B exciton emission of the MoS_2_ nanoscroll. The dots represent experimental data, and the solid lines are theoretical fits. Reproduced with permission from Ref. [79]. Copyright 2022 ACS.

Owing to their unique structure, TMDC nanoscrolls exhibit strong anisotropy. To investigate their anisotropic vibrational behavior, the Raman and PL spectra of a MoS_2_ nanosheet and a MoS_2_ nanoscroll were measured using polarization-dependent Raman spectroscopy at different angles. Figure 21 shows the polar plots of the peak intensities of the $E_{2g}^{1}$ and A_1g_ vibration modes, as well as the PL peaks, of a MoS_2_ nanosheet and a MoS_2_ nanoscroll [79]. The intensities of the $E_{2g}^{1}$ and A_1g_ Raman modes remain almost unchanged for the MoS_2_ nanosheet at various polarization angles (Figure 21a,c), indicating the isotropic structure of the monolayer MoS_2_ nanosheet. In contrast, the intensities of the $E_{2g}^{1}$ and A_1g_ modes for the MoS_2_ nanoscroll varied with a period of 180° as the polarization angle was changed. This variation in peak intensity reflects the influence of the nanoscroll axis orientations on Raman scattering. The intensity reached its maximum and minimum when the polarized laser was aligned with and perpendicular to the long axis of the nanoscroll (Figure 21b,d), respectively. Similarly, the intensities of the A and B exciton emissions also exhibited a period of 180° in the angle-resolved PL spectrum of the MoS_2_ nanoscroll (Figure 21e,f).

#### 4.2.5. Laser Emission

**Figure 22:**
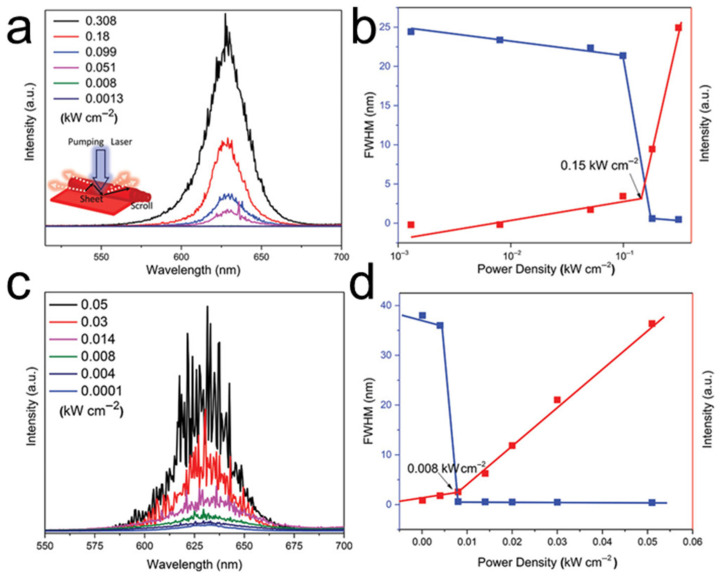
(**a**) High-resolution PL spectra of a WS_2_ nanoscroll (NS) excited by a pump laser at various power densities. (**b**) Full width at half maximum (FWHM) and PL intensity of the WS_2_ NS as a function of excitation power density. (**c**) PL spectra of a QD/WS_2_ NS excited by a pump laser at low excitation power density. (**d**) FWHM and PL intensity of the QD/WS_2_ NS as a function of excitation power density in the low-power regime. Reproduced with permission from Ref. [69]. Copyright 2020 Wiley.

Because a TMDC nanoscroll possesses a cylindrical microcavity structure, light is scattered randomly inside the microcavity under illumination. Owing to the difference in refractive index between the TMDC layer and air, total internal reflection occurs at the interface. Consequently, stimulated emission can be observed because light remains confined within the microcavity for a sufficiently long time. As shown in Figure 22a, the WS_2_ nanoscroll exhibited a similar PL peak to that of the WS_2_ nanosheet when irradiated by a low-power laser. However, when the laser pumping power exceeded a threshold of 0.15 kW/cm^2^, many weak but sharp peaks with a full width at half maximum (FWHM) of less than 1 nm appeared in the PL spectrum of the WS_2_ nanoscroll (Figure 22a,b). Interestingly, the threshold decreased significantly to 0.008 kW/cm^2^ when CdSe/ZnS quantum dots (QDs) were wrapped into the WS_2_ nanoscroll (denoted QD/WS_2_ NS) (Figure 22c,d). Furthermore, numerous strong lasing spikes were observed in the emission spectra of the QD/WS_2_ NS (Figure 22c). The strong random laser emission of the QD/WS_2_ NS can be attributed to Förster resonance energy transfer (FRET) between the CdSe/ZnS QDs and the 2D WS_2_, as well as to multiple scattering and reflection of light inside the nanoscroll [69].

### 4.3. Magnetoresistance

**Figure 23:**
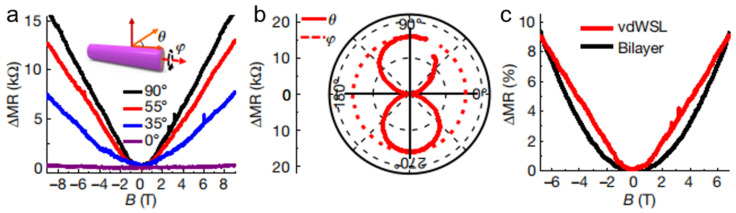
Magnetoresistance (ΔMR) of a SnS_2_/WSe_2_ nanoscroll. (**a**) Magnetoresistance (ΔMR) of the SnS_2_/WSe_2_ nanoscroll as a function of magnetic field at T = 3 K, measured at different rotation angles *θ*. (**b**) Angle-dependent magnetoresistance of the SnS_2_/WSe_2_ nanoscroll at a magnetic field of 9 T. (**c**) Magnetoresistance of the SnS_2_/WSe_2_ nanoscroll and nanosheet as a function of magnetic field at T = 3 K. Reproduced with permission from Ref. [40]. Copyright 2021 Springer Nature.

Zhao et al. investigated the magneto-transport properties of a SnS_2_/WSe_2_ hetero-bilayer nanoscroll [40]. The magnetoresistance of the SnS_2_/WSe_2_ hetero-bilayer nanoscroll was measured as a function of the rotation angles *θ* and *φ*, which are perpendicular and parallel to the nanoscroll axis (Figure 23a), respectively. The angle *θ* plays an important role in tuning the magnetoresistance magnitude. As shown in Figure 23a, the magnetoresistance magnitude decreased as *θ* decreased. In contrast, the magnetoresistance was insensitive to variations in *φ* owing to the rotational symmetry of the nanoscroll. As shown in Figure 23b, the magnetoresistance did not change with *φ* but exhibited a sine-function dependence on *θ* at *B* = 9 T [80], implying a 1D transport characteristic of the nanoscroll. Furthermore, the magnetoresistance of the 1D SnS_2_/WSe_2_ nanoscroll exhibited a clear linear relationship with the magnetic field at 3 K, whereas that of the 2D SnS_2_/WSe_2_ heterostructure showed a quadratic dependence (Figure 23c) [40]. The quadratic magnetoresistance dependence can be attributed to the Onsager reciprocity relation [81], as reported in other 1D and 2D systems. Linear magnetoresistance has previously been observed in gapless semiconductors and may arise from the topological properties of the band structure [82].

### 4.4. Electrical Properties

**Figure 24:**
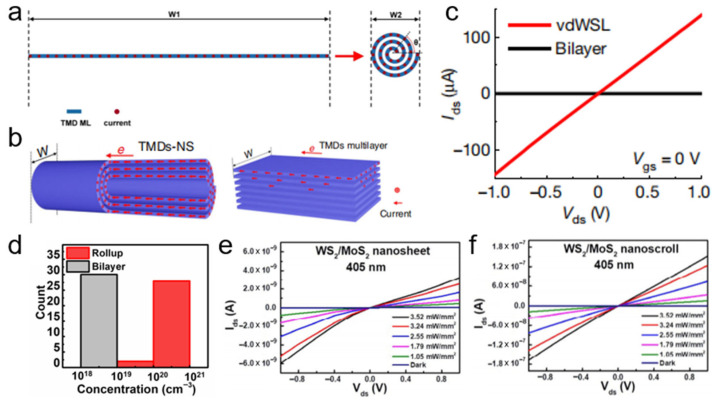
(**a**) Schematic illustration of the conduction channel width in a TMDC monolayer nanosheet and a TMDC nanoscroll. (**b**) Schematic of carrier transportation in a TMDC-nanoscroll and in TMDC multilayer. Reproduced from Ref. [17]. (**c**) Current of a FET based on a SnS_2_/WSe_2_ nanoscroll and a SnS_2_/WSe_2_ bilayer nanosheet. (**d**) Carrier density of a SnS_2_/WSe_2_ nanoscroll and a SnS_2_/WSe_2_ bilayer nanosheet. Reproduced with permission from Ref. [40]. Copyright 2021 Springer Nature. (**e**,**f**) I_ds_-V_ds_ curves of (**e**) a WS_2_/MoS_2_ nanosheet and (**f**) a WS_2_/MoS_2_ nanoscroll under 405 nm laser illumination at different power densities. Reproduced with permission from Ref. [12]. Copyright 2020 Springer Nature.

When a MoS_2_ nanosheet is rolled into a MoS_2_ nanoscroll, the width of the conduction channel is significantly reduced (Figure 24a) [17]. Consequently, the nanoscroll is expected to exhibit higher current density and mobility than the nanosheet. Furthermore, there are no charge traps or dangling bonds between adjacent layers of the MoS_2_ nanoscroll, which further improves carrier transport efficiency. Moreover, in a MoS_2_ nanoscroll, carriers are transported through the entire plane, whereas in a multilayer MoS_2_ nanosheet, they are confined to only a few shell layers (Figure 24b). Therefore, the MoS_2_ nanoscroll should exhibit higher mobility than both monolayer and multilayer MoS_2_ nanosheets. This explanation is supported by experimental data. Cui et al. found that the mobility of the MoS_2_ nanoscroll was in the range of 200–700 cm^2^ V^−1^ s^−1^, which is nearly 30 times higher than that of the MoS_2_ nanosheet [17]. Zhao et al. reported that the current of a field-effect transistor (FET) based on a SnS_2_/WSe_2_ nanoscroll was two to six orders of magnitude higher than that of an FET based on a SnS_2_/WSe_2_ nanosheet (Figure 24c) [40]. Furthermore, the carrier density of the SnS_2_/WSe_2_ nanoscroll was also two to three orders of magnitude higher than that of the SnS_2_/WSe_2_ nanosheet (Figure 24d).

Under illumination, TMDC nanoscrolls also exhibit excellent optoelectronic performance. The MoS_2_ nanosheet and nanoscroll have exciton lifetimes of 726 and 303 ps, respectively, as measured by time-resolved PL spectroscopy [79]. The MoS_2_ nanoscroll has shorter lifetime, indicating highly efficient separation of photogenerated charges. Owing to the anisotropic structure, photogenerated carriers preferentially transport along the nanoscroll axis. In contrast, in a MoS_2_ nanosheet, photogenerated carriers diffuse in all directions owing to its isotropic structure. Therefore, TMDC nanoscrolls are expected to exhibit faster electron transport than their nanosheet counterparts. Wang et al. found that the photocurrent of a WS_2_/MoS_2_ hetero-bilayer was 3.3 nA under 405 nm laser illumination [12]. In contrast, the photocurrent of a WS_2_/MoS_2_ nanoscroll was significantly enhanced to 152 nA under the same conditions (Figure 24e,f), demonstrating the excellent optoelectronic performance of TMDC nanoscrolls.

**Figure 25:**
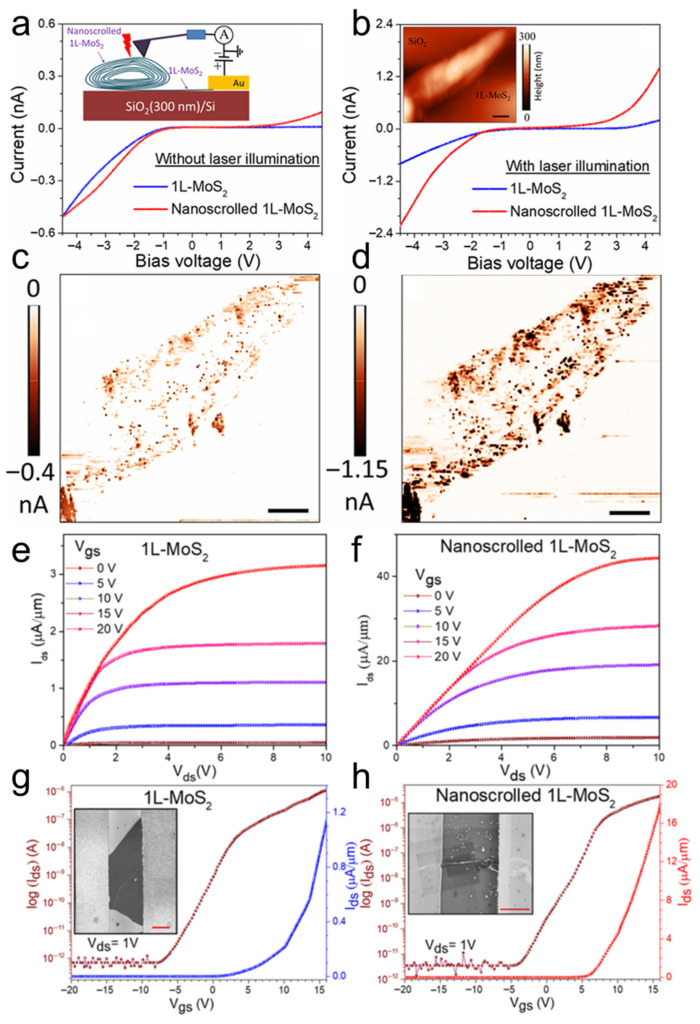
Electrical properties of MoS_2_ nanoscrolls and nanosheets. (**a**,**b**) I-V curves of (**a**) a MoS_2_ monolayer nanosheet and (**b**) a MoS_2_ nanoscroll under dark and illumination conditions, measured by C-AFM. (**c**,**d**) Current mapping images of a 1L-MoS_2_ nanoscroll under (**c**) dark and (**d**) illuminated conditions. Scale bar: 1 µm. (**e**,**f**) Output characteristics of (**e**) a MoS_2_ nanosheet and (**f**) a MoS_2_ nanoscroll. (**g**,**h**) Transfer curves of (**g**) a MoS_2_ nanosheet and (**h**) a MoS_2_ nanoscroll. Reproduced with permission from Ref. [83]. Copyright 2025 ACS.

By using conductive atomic force microscopy (C-AFM), Bhuyan et al. investigated in situ the photocurrents of a MoS_2_ nanoscroll and a MoS_2_ nanosheet [83]. The surface topography and electrical conductivity of nanomaterials can be obtained simultaneously by C-AFM at the nanoscale. As shown in Figure 25, the current distribution and *I*-*V* characteristics of a MoS_2_ nanoscroll and a MoS_2_ nanosheet were measured directly using C-AFM under dark and illuminated conditions, respectively. The MoS_2_ nanoscroll and nanosheet exhibited similar currents under dark conditions (Figure 25a), whereas the photocurrent of the MoS_2_ nanoscroll was 6 times higher than that of the MoS_2_ nanosheet (Figure 25b). The current distribution of the MoS_2_ nanoscroll and nanosheet was mapped by point-by-point current measurements on the sample surface (Figure 25c,d). The current of the MoS_2_ nanoscroll increased under illumination, indicating that more photogenerated carriers were generated than under dark conditions. In addition, they found that the MoS_2_ nanoscroll-based FET device showed a 15-fold higher saturated photocurrent than the MoS_2_ nanosheet-based FET device at the same conditions (Figure 25e,f). The MoS_2_ nanoscroll-based FET device also exhibited an on/off current ratio one order of magnitude higher than that of the MoS_2_ nanosheet-based FET device (Figure 25g,h). Importantly, the MoS_2_ nanoscroll-based FET device exhibited a mobility of approximately 2400 ± 400 cm^2^ V^−1^ s^−1^, which was about 2 orders of magnitude higher than that of the MoS_2_ nanosheet-based FET device (approximately 10.5 cm^2^ V^−1^ s^−1^). These results indicate that the electrical performance of the MoS_2_ nanoscroll is substantially better than that of the MoS_2_ nanosheet.

### 4.5. Phase Transition

Compared with 2H-phase TMDC nanosheets, 1T-phase nanosheets exhibit dramatically reduced charge transfer resistance owing to their metallic phase structure, which has attracted intensive attention in the fields of energy storage and hydrogen evolution reactions. However, 1T-phase TMDC is metastable and is easily converted to the stable 2H-phase. Therefore, obtaining stable 1T-phase TMDC at room temperature remains a challenge.

**Figure 26:**
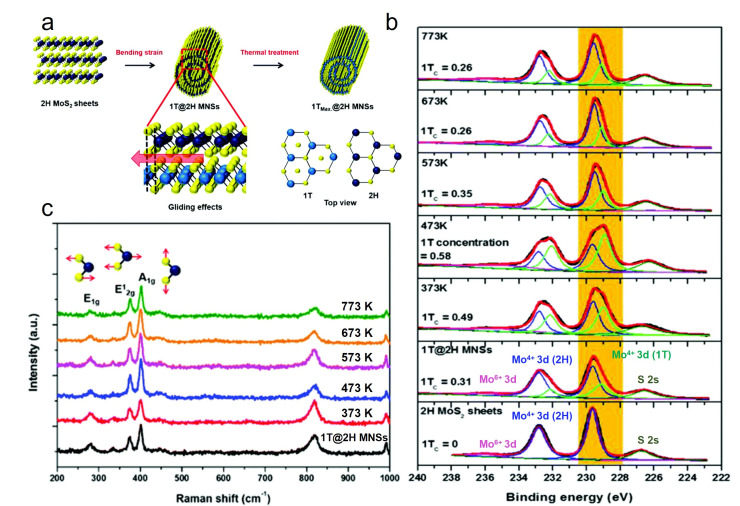
Phase transition from 2H to 1T in MoS_2_ nanoscrolls. (**a**) Schematic illustration of the transition from 2H-MoS_2_ nanosheets to 1T@2H mixed-phase MoS_2_ nanoscrolls (MNSs) via scrolling and thermal treatment. (**b**) Mo 3d XPS spectra of 1T@2H MNSs heated at different temperatures. (**c**) Raman spectra of 1T@2H MNSs heated at different temperatures. Reproduced with permission from Ref. [53]. Copyright 2017 RSC.

In 2017, Hwang and Suh reported the phase transition from 2H-MoS_2_ to 1T-MoS_2_ during the formation of MoS_2_ nanoscrolls in solution. As the MoS_2_ nanosheet was rolled up, lattice distortion induced intra-layer plane gliding. Consequently, the 1T phase formed upon movement of the intra-layer S plane (Figure 26a). The proportion of the 1T phase increased with increasing uniaxial bending strain as the MoS_2_ nanosheet continued to scroll.

X-ray photoelectron spectroscopy (XPS) was employed to analyze the composition and chemical state of the MoS_2_ nanoscroll. As shown in Figure 26b, the 2H-MoS_2_ nanosheet exhibited two peaks at 229.5 and 233 eV, which were attributed to the Mo^4+^ 3d_5/2_ and Mo^4+^ 3d_3/2_, respectively. Upon deconvolution of the Mo 3d region in the high-resolution XPS spectra, additional peaks at approximately 232 and 228.9 eV appeared, arising from the 1T-phase MoS_2_ and confirming the coexistence of 1T and 2H phases. Furthermore, the proportion of 1T phase can be obtained by analyzing the area of the corresponding peaks. Upon annealing the MoS_2_ nanoscroll from room temperature to 773 K, the proportion of the 1T phase first reached a maximum of 0.58 at 473 K and then decreased to 0.26 as the temperature further increased to 673 K. For a 1T-phsae MoS_2_ nanosheet prepared by exfoliating MoS_2_ powder in *n*-butyl lithium, 90% of the 1T phase was converted to the 2H phase at 473 K. The stability of the 1T phase in the MoS_2_ nanoscroll at elevated temperatures was also confirmed by Raman spectroscopy. As shown in Figure 26c, the two dominant peaks at 381 and 406 cm^−1^ were attributed to the 2H-phase MoS_2_. However, an E_1g_ mode peak at 274 cm^−1^ was observed in the Raman spectrum of the MoS_2_ nanoscroll; this peak is inactive in 2H-phase MoS_2_, confirming the presence of the 1T phase in the nanoscroll [53,84].

This section presents the unique properties of TMDC nanoscrolls in terms of morphology, optics, electrical transport, magnetic transport, and phase transition. A thorough investigation of these properties enables a better understanding of how the nanoscroll structure influences device performance, thereby guiding its practical applications.

AFM and TEM characterizations show that TMDC nanoscrolls have a compact spiral structure, with an interlayer spacing of approximately 0.69 nm, which matches the monolayer thickness, indicating a closely packed structure. Second harmonic generation (SHG) studies reveal that the rolling angle (*θ*_roll_) directly determines the degree of SHG signal enhancement (up to 95 times that of monolayer nanosheets) and the nonlinear chiral response (continuously tunable SHG circular dichroism). The enhanced SHG signal originates from symmetry breaking and chirality generation induced by rolling. Raman spectra show that the A1g peak of nanoscrolls generally exhibits a blue shift due to enhanced interlayer coupling, whereas the E_2g_ peak displays a red shift caused by uniaxial strain from bending. Moreover, the number and polarization response of low-frequency breathing (LB) modes in ultra-low-frequency Raman spectra directly reflect the compactness of interlayer stacking. MoS_2_ nanoscrolls prepared by the water-dragging method exhibit more LB peaks than those prepared by evaporating an ethanol droplet, confirming that the solvent-free strategy yields a more compact structure. In photoluminescence (PL) spectra, tightly rolled nanoscrolls usually show a red shift resulting from strain and stacking effects, whereas loosely structured nanoscrolls exhibit anomalous blue shifts accompanied by enhanced PL intensity.

MoS_2_ nanoscrolls prepared using ethanol exhibit a significantly higher dark current, indicating that residual ethanol molecules weaken interlayer coupling. Marangoni flow induced by organic solvent evaporation frequently generates inhomogeneous strain in TMDC nanoscrolls, leading to local intensity variations in SHG mapping. In contrast, heterojunction nanoscrolls (e.g., SnS_2_/WSe_2_) fabricated by alkaline solution etching display angle-dependent magnetoresistance, implying a uniform scrolling structure. MoS_2_ nanoscrolls prepared through LCA self-assembly show characteristic 1T phase signatures in XPS and Raman spectra, demonstrating that rolling-induced lattice sliding can stabilize the metastable 1T phase. Nanoscrolls obtained by plasma treatment commonly present an amorphous structure with significantly broadened Raman peaks. In contrast, nanoscrolls synthesized via the supercritical fluid method and VFD shearing method maintain excellent crystallinity.

More importantly, the same characterization result may originate from different microscopic mechanisms. For example, a PL red shift could correspond to either strain or stacking-induced changes in the indirect bandgap, requiring differentiation with the aid of theoretical simulations. Furthermore, high-resolution characterization of the internal defect distribution in nanoscrolls—such as the orientation of sulfur vacancies along the rolling direction—remains lacking at present.

## 5. Applications

Owing to their high mobility, thickness-dependent band structure, van der Waals interfaces, and excellent mechanical flexibility, TMDC nanosheets have become ideal materials for photodetectors. Compared with monolayer TMDC nanosheets, TMDC nanoscrolls exhibit an enlarged cross-section, which improves light absorption. In addition, light–matter interaction in TMDC nanoscrolls is enhanced owing to total internal reflection within their scrolled structure. Moreover, strain at the curved interface of the scroll induces bandgap variation and enhances light–matter coupling. Therefore, the optoelectronic performance of TMDC nanoscrolls is significantly improved compared with that of monolayer TMDC nanosheets. Owing to the excellent physical and chemical properties arising from their unique structure, TMDC nanoscrolls are extensively explored in optoelectronics, catalytic hydrogen evolution, gas sensing, and synaptic applications. In this section, we discuss the latest progress of TMDC nanoscrolls in various applications.

### 5.1. Photodetector

To evaluate the optoelectronic performance of a photodetector, the representative parameters are PDR (photocurrent-to-dark-current ratio), R (photoresponsivity), EQE (external quantum efficiency), and D* (detectivity). Their definitions are as follows.

(2) $$\mathrm{PDR}=I_{\mathrm{photo}}/I_{\mathrm{dark}}$$

where I_photo_ is photocurrent, and I_dark_ is dark current.

(3) $$R=I_{\mathrm{photo}}/\mathrm{PS}$$

where P is the laser power density, and S is the effective area of the device.

(4) EQE= hcR/eλ

where h is the Planck’s constant, c is the speed of light, e is the charge, and λ is the laser wavelength.


(5) $$D^{*}=RS^{1/2}/(2eI_{\mathrm{dark}}{)}^{1/2}$$

#### 5.1.1. Photodetectors Based on TMDC Nanoscrolls

When 2D TMDC nanosheets are transformed into 1D nanoscrolls, their tubular structures facilitate the movement of photogenerated carriers along the axis. In addition, strain from the curvature of the scroll induces bandgap variation. Therefore, TMDC nanoscrolls are promising candidates for photodetectors.

**Figure 27:**
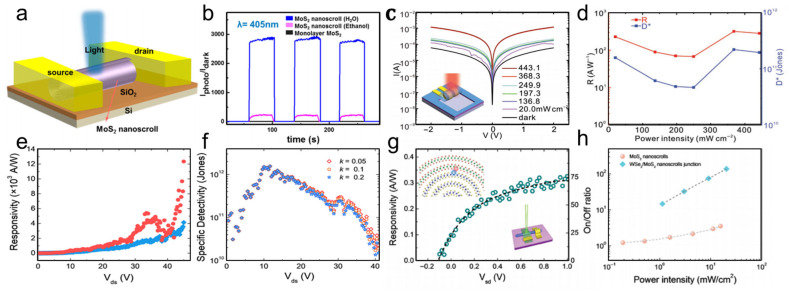
Photodetectors based on TMDC Nanoscrolls. (**a**) Schematic of a MoS_2_ nanoscroll photodetector. (**b**) PDRs comparison of MoS_2_ nanosheet, MoS_2_ NS-W, and MoS_2_ NS-E photodetectors under 405 nm laser. Reproduced with permission from Ref. [39]. Copyright 2022 ACS. (**c**) I-V curves of MoSe_2_ nanoscroll device in dark and under 808 nm laser. (**d**) Responsivity and detectivity of MoSe_2_ nanoscroll device versus laser power. Reproduced with permission from Ref. [21]. Copyright 2019 Wiley. (**e**) Responsivity and (**f**) detectivity of MoS_2_ nanoscroll avalanche photodetector versus bias voltage. Adapted with permission from Ref. [15]. Copyright 2020 ACS. (**g**) Responsivity and EQE of WSe_2_/MoS_2_ nanoscroll under 532 nm laser. (**h**) On/off ratios of WSe_2_/MoS_2_ and MoS_2_ nanoscroll devices versus light power. Reproduced with permission from Ref. [86]. Copyright 2019 Wiley.

Zhao et al. investigated the optoelectronic performance of a monolayer MoS_2_ nanosheet and a MoS_2_ nanoscroll by comparing their PDRs (Figure 27a) [39]. The MoS_2_ nanoscrolls were prepared by dropping an ethanol droplet and dragging a water droplet across the CVD-grown monolayer MoS_2_ nanosheets, respectively, and are referred to as MoS_2_ NS-E and MoS_2_ NS-W. Under 405 nm laser illumination, the PDR of the monolayer MoS_2_ nanosheet was approximately 6, whereas the PDRs of MoS_2_ NS-E and MoS_2_ NS-W were 230 and 2800 (Figure 27b), respectively. The enhanced PDR of MoS_2_ NS-W can be explained as follows. Although MoS_2_ NS-W had a comparable photocurrent to MoS_2_ NS-E, the dark current of MoS_2_ NS-W was one order of magnitude lower than that of MoS_2_ NS-E [39]. For MoS_2_ NS-E, ethanol residues were inevitably wrapped between the adjacent layers of the nanoscroll during the rapid preparation process, which can donate electrons to the MoS_2_ layer, increasing the dark current of the TMDC nanoscrolls [39]. Under illumination, the encapsulated ethanol residues further reduced light absorption efficiency. Compared with MoS_2_ NS-W, which contained no solvent residue, less light passed through the ethanol/MoS_2_ layer. Moreover, the presence of ethanol hindered interlayer transport of photogenerated carriers. Therefore, the PDR of MoS_2_ NS-W was one order of magnitude higher than that of MoS_2_ NS-E.

Zhou et al. fabricated a photodetector based on MoSe_2_ scrolls using a self-rolled-up technique [21]. The as-prepared MoSe_2_ scroll exhibited a lower dark current than the planar MoSe_2_ film. At the same bias voltage, the dark current of the planar MoSe_2_ film (1.13 × 10^−4^ A) was approximately 2 times that of the MoSe_2_ scroll (6.03 × 10^−5^ A). The reduced dark current can be attributed to the following factors. Owing to the curvature effect, a uniaxial tensile strain of approximately 3.2% developed in the MoSe_2_ scroll, which increased the Schottky barrier at the electrode–MoSe_2_ interface. Consequently, the number of carriers injected from the source was reduced owing to the increased barrier. In addition, the rolled-up structure exposed a larger surface area to air than the planar structure, resulting in a higher density of surface states that can trap charge and reduce the effective conductive region. When the devices were illuminated with an 808 nm laser at the same optical power density, the photocurrent of the MoSe_2_ scroll was one order of magnitude higher than that of the planar MoSe_2_ film. Under illumination from an 808 nm laser with a power density of 368.3 mW/cm^2^, the MoSe_2_ scroll exhibited a responsivity (R) of 282.5 A/W, a detectivity (D*) of 1.96 × 10^11^ Jones, and a photosensitivity (PDR) of 19—values that were greatly enhanced compared with those of the planar MoSe_2_ film (Figure 27c,d).

Owing to the avalanche multiplication effect, numerous secondary electron–hole pairs are accelerated to extremely high kinetic energies in a strong electric field [85], resulting in a carrier multiplication process. Consequently, the performance of optoelectronic devices can be greatly enhanced through significant amplification of the number of photogenerated carriers. Deng et al. demonstrated the avalanche multiplication effect in a MoS_2_ nanoscroll-based photodetector [15]. In a MoS_2_ nanoscroll, the band gap is reduced and scattering is suppressed owing to the strain effect. As a result, avalanche multiplication can be triggered by a significantly lower electric field compared with that required for a MoS_2_ nanosheet. Consequently, the MoS_2_ nanoscroll exhibited an ultrahigh photoresponsivity of >10^4^ A/W and a specific detectivity of approximately 2 × 10^12^ Jones (Figure 27e,f). Using a dry-transfer method, they also integrated a mechanically exfoliated WSe_2_ nanosheet with a MoS_2_ nanoscroll, thereby forming a heterojunction (Figure 27g). The resulting heterojunction enhanced the built-in potential, which in turn reduced the surface recombination effect. As a result, the dark current was effectively suppressed to less than 10^−12^ A, and the PDR exceeded 10^2^—approximately two orders of magnitude higher than that of a single MoS_2_ nanoscroll (Figure 27h). At a bias voltage of 1 V, the photoresponsivity of the WSe_2_/MoS_2_ NS device reached 0.3 A/W, and the external quantum efficiency was approximately 75% (Figure 27g). The response time of the WSe_2_/MoS_2_ NS device was only 5 ms, which was approximately three orders of magnitude faster than that of a photodetector based on a MoS_2_ nanoscroll [86].

#### 5.1.2. Photodetectors Based on TMDC Nanoscrolls Encapsulating with Photosensitive Nanomaterials

Owing to their atomically thin thickness, single-layer TMDC nanosheets absorb less than 10% of visible light [87]. Incorporating photosensitive materials such as nanoparticles, quantum dots, and organic dyes has been proposed as an effective strategy to enhance the light absorption of TMDC nanosheets. Therefore, encapsulating photosensitive nanomaterials within TMDC nanoscrolls is also considered a promising approach to further improve optoelectronic performance.

**Figure 28:**
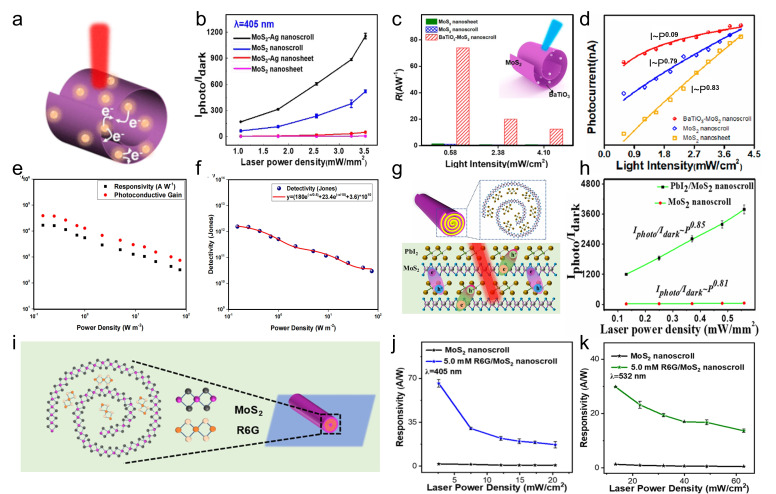
Photodetectors based on TMDC Nanoscrolls encapsulating photoactive nanomaterials. (**a**) Schematic diagram of MoS_2_-Ag NS. (**b**) PDR curves of a MoS_2_-Ag NS, a MoS_2_ NS, a MoS_2_-Ag nanosheet, and a MoS_2_ nanosheet as a function of laser power density under 405 nm laser illumination. Reproduced with permission from Ref. [74]. Copyright 2021 ACS. (**c**) Histogram of the responsivity and (**d**) photocurrent curves of a MoS_2_ nanosheet, a BaTiO_3_-MoS_2_ nanoscroll, and a MoS_2_ nanoscroll as a function of light power density. Reproduced with permission from Ref. [42]. Copyright 2023 ACS. (**e**) Responsivity and photoconductive gain, and (**f**) detectivity of a QD/WS_2_ nanoscroll as a function of light power density. Reproduced with permission from Ref. [69]. Copyright 2020 Wiley. (**g**) Schematic diagram of a PbI_2_-MoS_2_ nanoscroll. (**h**) PDR curves of a PbI_2_/MoS_2_ nanoscroll and a MoS_2_ nanoscroll as a function of laser power density. Reproduced with permission from Ref. [88]. Copyright 2022 ACS. (**i**) Schematic diagram of an R6G/MoS_2_ nanoscroll. Responsivity of a MoS_2_ nanoscroll and an R6G/MoS_2_ nanoscroll as a function of laser power density under (**j**) 405 nm and (**k**) 532 nm laser illumination. Reproduced from Ref. [91].

In 2021, Yue et al. reported the encapsulation of silver (Ag) nanoparticles (NPs) within MoS_2_ and WS_2_ nanoscrolls to improve the photoresponse [74]. MoS_2_ and WS_2_ nanosheets were first grown on a SiO_2_/Si substrate by CVD; then, a silver nitrate (AgNO_3_) solution was spin-coated onto them to obtain Ag-nanoparticle-decorated MoS_2_ and WS_2_ nanosheets (referred to as MoS_2_-Ag and WS_2_-Ag nanosheets). By dropping alkaline solution onto the MoS_2_-Ag and WS_2_-Ag nanosheets at 60 °C, MoS_2_-Ag and WS_2_-Ag nanoscrolls were obtained [74]. Under blue light (405 nm) irradiation, the PDR of the MoS_2_-Ag nanoscroll was significantly improved compared with that of a MoS_2_ nanosheet (Figure 28a). Due to the encapsulation of Ag NPs, the PDR of MoS_2_-Ag nanoscroll was approximately twice that of a MoS_2_ nanoscroll. When Ag NPs were encapsulated in the nanoscroll, incident light scattering and near-field oscillations of conducting electrons were enhanced by the increased local electrical field and light absorption induced by the Ag NPs [74]. In addition, the multilayer structure of the nanoscroll further increased the light absorption cross-section. Moreover, the MoS_2_ layers of the nanoscroll could accept electrons from the Ag NPs decorating both sides, further increasing the photocurrent. Consequently, the MoS_2_-Ag and WS_2_-Ag nanoscrolls exhibited much higher PDRs than the MoS_2_ and WS_2_ nanosheets, as well as the MoS_2_ and WS_2_ nanoscrolls (Figure 28b).

Su et al. prepared a BaTiO_3_/MoS_2_ nanoscroll by wrapping BaTiO_3_ nanoparticles into a MoS_2_ nanoscroll using NaHCO_3_ solution at 60 °C [42]. Under illumination from a 470 nm laser with a power density of 0.58 mW/cm^2^, the photoresponsivity of the BaTiO_3_/MoS_2_ nanoscrolls was 73.9 A/W—much higher than those of a MoS_2_ nanoscroll and a MoS_2_ nanosheet (Figure 28c,d). After CdSe-ZnS core–shell quantum dots (QDs) were deposited onto a CVD-grown monolayer WS_2_ nanosheet by spin-coating, Ghosh et al. prepared a QD/WS_2_ hybrid nanoscroll using volatile acetone solvent [69]. A p-n like junction formed between the QDs and the WS_2_ layer in the QD/WS_2_ nanoscroll, enabling effective separation of photogenerated carriers. Meanwhile, recombination of electron-hole pairs was suppressed. Consequently, the separation of electron–hole pairs was accelerated, generating an ultrahigh photocurrent. As a result, the photoresponsivity, photo gain, and detectivity were greatly enhanced to 1.67 × 10^4^ A/W, 3.9 × 10^4^, and 1.5 × 10^12^ Jones (Figure 28e,f), respectively—the highest values among NS-based devices at that time.

In 2022, Wu et al. immersed CVD-grown monolayer MoS_2_ nanosheets in a PbI_2_/DMF solution to obtain PbI_2_/MoS_2_ nanosheets. Subsequently, PbI_2_/MoS_2_ nanoscrolls were successfully prepared by dropping a mixed solution of ammonia and isopropanol onto the PbI_2_/MoS_2_ nanosheets at 80 °C [88]. Multiple type-II heterojunction interfaces formed between the PbI_2_ and MoS_2_ layer, which promoted the generation and separation of photogenerated carriers, thereby greatly improving the optoelectronic performance of the PbI_2_/MoS_2_ nanoscroll (Figure 28g). Under 405 nm, 532 nm, and 633 nm lasers, the PDRs of the PbI_2_/MoS_2_ nanoscroll were 48,185, 3670, and 25,662, respectively, which were three orders of magnitude higher than those of a MoS_2_ nanosheet. Compared with a MoS_2_ nanoscroll, the PDR of the PbI_2_/MoS_2_ nanoscroll was also increased by two orders of magnitude. Under 532 nm laser, the PDRs of the PbI_2_/MoS_2_ nanoscroll were substantially higher than those of a MoS_2_ nanoscroll across various light power densities (Figure 28h).

As a photoactive organic dye, rhodamine (R6G) has been widely used to enhance the photoresponsivity and detectivity of optoelectronic devices [89,90]. In 2024, Ye et al. encapsulated R6G within MoS_2_ (R6G/MoS_2_) nanoscrolls to enhance light absorption and photoresponse. In the resulting R6G/MoS_2_ nanoscrolls, multiple type-II heterojunction interfaces played a crucial role in facilitating photogenerated carriers and the subsequent separation of electron–hole pairs (Figure 28i). The separated electrons were transported rapidly along the nanoscroll axis. Under a 405 nm laser, the photoresponsivity of the R6G/MoS_2_ nanoscrolls was four orders of magnitude higher than that of a single-layer MoS_2_ nanosheet [91] (Figure 28j,k). The R6G/MoS_2_ nanoscrolls maintained good optoelectronic performance even after 6 months, whereas the photoresponsivity of R6G/MoS_2_ nanosheets decreased significantly. This clearly indicates that the nanoscroll structure can effectively protect R6G from degradation under ambient conditions, thereby maintaining its excellent optoelectronic performance.

Table 3 summarizes the impact of various encapsulated nanomaterials on the performance of TMDC nanoscrolls.

#### 5.1.3. Polarization Sensitive Photodetector

**Figure 29:**
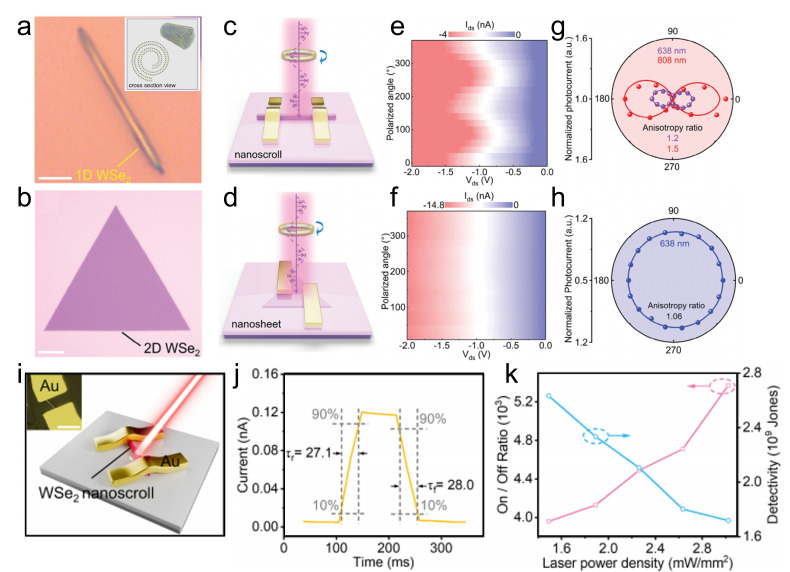
Polarization-sensitive photodetectors based on bilayer WSe_2_ nanoscrolls. (**a**) OM image of a bilayer WSe_2_ nanoscroll. Adapted with permission from Ref. [19]. Copyright 2024 Wiley. (**b**) OM image of a triangular bilayer WSe_2_ nanosheet. (**c**,**d**) Device schematics of WSe_2_ nanoscroll (**c**) and nanosheet (**d**) under polarized light. (**e**,**f**) Bias-dependent angular-resolved photocurrent maps for WSe_2_ nanoscroll (**e**) and nanosheet (**f**) devices. (**g**,**h**) Polar plots of normalized photocurrent for WSe_2_ nanoscroll (**g**) and nanosheet (**h**) under 638/808 nm polarized light. Reproduced with permission from Ref. [19]. Copyright 2024 Wiley. (**i**) Schematic configuration, (**j**) time-resolved photoresponse, and (**k**) detectivity and on/off ratio of a bilayer WSe_2_ nanoscroll-based photodetector. Reproduced with permission from Ref. [92]. Copyright 2024 IOP.

Ao et al. investigated the photoresponse of a WSe_2_ nanoscroll transformed from a bilayer WSe_2_ nanosheet (Figure 29a,b) [19]. The photocurrent of the WSe_2_ nanoscroll was sensitive to the polarization direction of the incident light, in contrast to that of the WSe_2_ nanosheet. The WSe_2_ nanoscroll exhibited maximal and minimal photocurrents when the polarization direction was parallel and perpendicular to the nanoscroll axis (Figure 29c–e), respectively. In contrast, the photocurrent of the bilayer WSe_2_ nanosheet did not exhibit any periodic change as the polarization direction varied (Figure 29f–h). Under 808 nm laser illumination, the anisotropy photocurrent ratio of the WSe_2_ nanoscroll reached 1.5 (Figure 29e), demonstrating the potential of TMDC nanoscrolls as polarization-sensitive photodetectors. Lan et al. found that the photodetector based on a bilayer WSe_2_ nanoscroll exhibited an excellent response in the spectral range of 365–830 nm. It exhibited a rise time of 27.1 ms, a fall time of 28 ms, an on-off ratio as high as 5.3 × 10^3^, an ultralow dark current of 5 × 10^−14^ A, and a detectivity of 2.63 × 10^9^ Jones (Figure 29i–k). The aforementioned excellent optoelectronic performance is attributed to the increased carrier concentration in the bilayer nanoscroll, the strain-enhanced light-matter interaction, multiple light reflections in the nanoscroll microcavities, and the accelerated charge-carrier mobility along the 1D nanoscroll axis [92].

**Figure 30:**
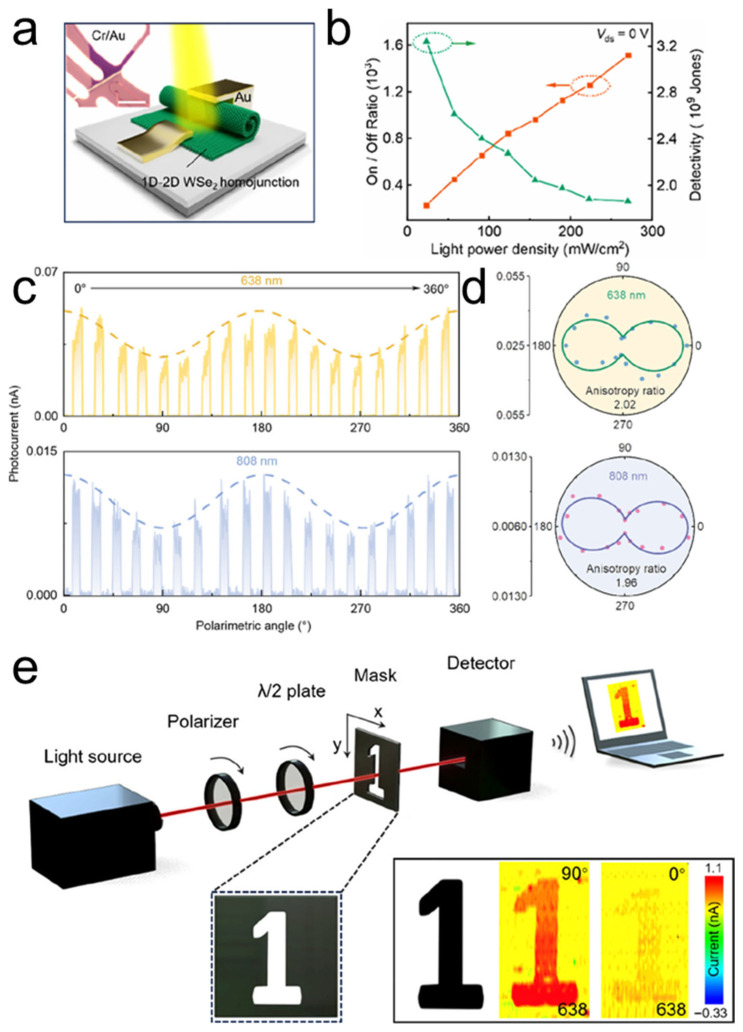
Polarization-sensitive photodetector based on a 1D WSe_2_ nanoscroll/2D WSe_2_ nanosheet homojunction. (**a**) Schematic of a photodetector based on the 1D/2D WSe_2_ homojunction. (**b**) On/off ratio and detectivity as a function of light power density at V_ds_ = 0 V. (**c**) Polarization-dependent photocurrent under illumination of (**top**) 638 nm and (**bottom**) 808 nm lasers. (**d**) Polar coordinates of the angular-resolved normalized photocurrent under 638 nm and 808 nm laser illumination. (**e**) Schematic setup for polarimetric imaging, along with measured images without a polarizer or with a polarizer at 90° and 0° through a mask under 638 nm laser illumination. Reproduced with permission from Ref. [77]. Copyright 2024 ACS.

In 2024, Zhang et al. reported a high-performance, self-powered, polarization-sensitive photodetector based on a 1D WSe_2_ nanoscroll/2D WSe_2_ nanosheet (1D/2D WSe_2_) homojunction [77]. By precisely controlling the volume ratio of ethanol to water, a WSe_2_ nanosheet was partially rolled up to form a 1D/2D WSe_2_ homojunction structure in the ethanol/water solution (Figure 30a). The as-fabricated device exhibited excellent self-powered optoelectronic performance, including a photoresponse across a wide spectral range of 405–808 nm, an on/off ratio of 1.5 × 10^3^, and a detectivity of 3.24 × 10^9^ Jones (Figure 30b). This performance was attributed to the ultralow dark current at zero bias voltage. The 1D/2D WSe_2_ homojunction also showed a polarization-dependent photoresponse. As the polarization angle of the incident light varied, maximal photocurrent values were observed at 0° and 180°, whereas minimal values were observed at 90° and 270° under both 638 and 808 nm laser illumination (Figure 30c). In addition, the homojunction exhibited dichroic ratios of 2.02 and 1.96 under 638 and 808 nm laser illumination (Figure 30d), respectively, indicating strong polarization sensitivity. The 1D/2D WSe_2_ homojunction also exhibited good imaging capability under polarized light. As shown in Figure 30e, an imaging detection platform based on the homojunction presented a clear difference under light with polarization angles of 0° and 90°, indicating its excellent multispectral and polarization-sensitive imaging capabilities.

### 5.2. Miniaturized Memory

**Figure 31:**
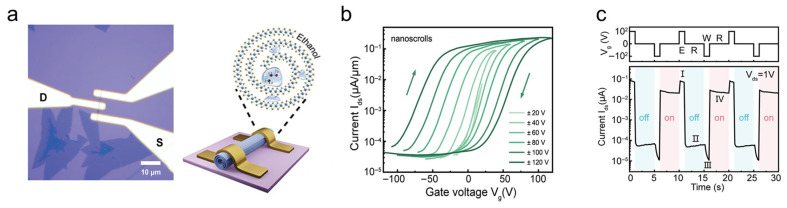
Miniaturized memory based on an individual MoS_2_ nanoscroll. (**a**) Optical image of a memory device based on a MoS_2_ nanoscroll. (**b**) Transfer characteristics of a MoS_2_ nanoscroll-based transistor at a bias voltage of 1 V. (**c**) Current-time plot of a MoS_2_ nanoscroll device under repetitive input gate voltage pulses at a bias voltage of 1 V. Reproduced with permission from Ref. [35]. Copyright 2024 ACS.

Using an improved volatile organic solvent evaporation-induced curling method, Qiao et al. prepared MoS_2_ nanoscrolls with high yield and good axial uniformity (Figure 31a) [35]. A field-effect transistor was then fabricated using the as-prepared MoS_2_ nanoscroll as the channel material. Owing to ethanol molecules trapped between adjacent layers, the nanoscroll transistor exhibited a large hysteresis during cyclic gate voltage (V_g_) scanning, and the on/off ratio was significantly reduced (Figure 31b). The width of the hysteresis window increased with the scanning amplitude of V_g_ and remained highly repeatable after multiple scanning cycles. The maximum window width reached 131 V when V_g_ was scanned from −120 V to 120 V.

To demonstrate the memory functionality, a series of V_g_ pulses with a width of 1 s were applied to program the conductance states of the MoS_2_ nanoscroll-based FET (Figure 31c). The MoS_2_ nanoscroll was capacitively charged when a V_g_ pulse of 100 V was applied (marked as “I” in Figure 31c). In this case, some electrons were driven into trap states, corresponding to the Erase process (marked as “E” in Figure 31c). The MoS_2_ nanoscroll was modulated to a high-resistance state when a V_g_ pulse of 0 V was applied (marked as “II” in Figure 31c), releasing the capacitive charge and corresponding to the Read process (marked as “R” in Figure 31c). The electrons were further depleted in the channel when a V_g_ pulse of −100 V was applied (marked as “III” in Figure 31c), corresponding to the Write process (marked by “W” in Figure 31c). Thus, the complete “Erase-Read-Write-Read” sequence was demonstrated, enabling multi-level memory storage. The MoS_2_ nanoscroll still exhibited an on/off ratio of approximately 10 even after a retention time of 1000 s, indicating the stability of the resistance state. In the charge-accumulation state, the curved surface of the MoS_2_ nanoscroll could generate a strong radial electric field, which facilitated charge trapping into the surrounding ethanol filler (which has a high dielectric constant), thereby enabling efficient local charge storage. Owing to the small volume of the 1D MoS_2_ nanoscroll, high-speed writing and erasing operations can be realized by precisely modulating a tiny number of charges, enabling miniaturized memory.

### 5.3. Electrocatalytic Hydrogen Evolution Reaction

**Figure 32:**
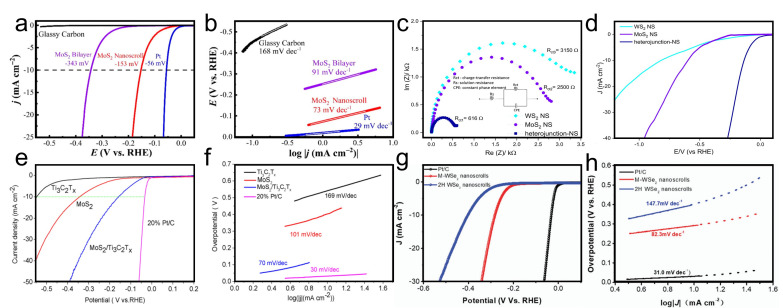
Electrocatalytic hydrogen evolution reaction (HER) performance of TMDC nanoscrolls. (**a**) Linear sweep voltammetry (LSV) polarization curves for HER and (**b**) Tafel slopes of glassy carbon, a bilayer MoS_2_, a MoS_2_ nanoscroll, and Pt electrodes in 0.5 M H_2_SO_4_. Reproduced with permission from Ref. [93]. Copyright 2019 ACS. (**c**) Nyquist plots and (**d**) LSV curves of a WS_2_ nanoscroll, a MoS_2_ nanoscroll, and their heterojunction nanoscroll in 0.5 M H_2_SO_4_. Reproduced with permission from Ref. [32]. Copyright 2022 ACS. (**e**) LSV curves and (**f**) Tafel plots of Ti_3_C_2_T_x_, MoS_2_, MoS_2_/Ti_3_C_2_T_x_, and 20% Pt/C in 0.5 M H_2_SO_4_. Reproduced with permission from Ref. [94]. Copyright 2019 Elsevier. (**g**) LSV curves and (**h**) Tafel plots of Pt/C, an M-WSe_2_ nanoscroll, and a 2H-WSe_2_ nanoscroll in 0.5 M H_2_SO_4_. Reproduced with permission from Ref. [95]. Copyright 2022 RSC.

The hydrogen evolution reaction plays an important role in the fields of energy, environmental, and chemical engineering. Transforming 2D TMDC nanosheets into 1D TMDC nanoscrolls exposes more active sites at the curled edges, thereby enhancing electrocatalytic performance [52,93]. In a TMDC nanoscroll, the potential barrier between adjacent layers is lower than that in a TMDC nanosheet, owing to increased interlayer coupling. Consequently, electrons can be easily transported from the glassy carbon electrode to the active sites, resulting in excellent HER performance. Jiang et al. prepared bilayer MoS_2_ nanoscrolls by immersing bilayer MoS_2_ nanosheets on a SiO_2_/Si substrate in hot KOH solution. The as-prepared MoS_2_ nanoscroll exhibited an overpotential of −153 mV at −10 mA·cm^−2^, and a Tafel slope of 73 mV/dec (Figure 32a,b) [93]. In contrast, the overpotential and Tafel slope of the bilayer MoS_2_ nanosheet were −343 mV and 91 mV/dec, respectively. Thus, the bilayer MoS_2_ nanoscroll exhibited much better HER catalytic performance than the bilayer MoS_2_ nanosheet.

Ghosh et al. found that WS_2_/MoS_2_ heterojunction nanoscrolls not only improved photogenerated carrier generation efficiency but also enhanced electrocatalytic efficiency [32]. Electrochemical impedance spectroscopy measurements indicated that the WS_2_/MoS_2_ heterojunction nanoscroll had a much lower charge-transfer resistance (616 Ω) than those of the MoS_2_ nanoscroll (2500 Ω) and the WS_2_ nanoscroll (3150 Ω) (Figure 32c), implying enhanced HER performance of the heterojunction nanoscroll. At a current density of 2 mA·cm^−2^, the overpotential of heterojunction nanoscroll was 50 mV, and the Tafel slope was 111 mV·dec^−1^ (Figure 32d). Moreover, the WS_2_/MoS_2_ heterojunction nanoscroll exhibited good electrochemical stability even after 72 h.

Janardhanan et al. synthesized MoS_2_ nanosheets using a hydrothermal method [14]. After a phenothiazine-based dye (PT) was adsorbed on the MoS_2_ nanosheets, MoS_2_/PT nanoscrolls were obtained through a self-assembly process facilitated by non-covalent interactions between the MoS_2_ nanosheets and PT. The number of active edge sites on MoS_2_ exposed to the electrode increased because of PT adsorption, thereby enhancing HER performance. The MoS_2_ nanosheet exhibited an overpotential of 432 mV and a Tafel slope of 184 mV·dec^−1^. In contrast, the MoS_2_/PT nanoscroll exhibited an overpotential of 343 mV and a Tafel slope of 141 mV·dec^−1^ [14].

Liu et al. added ammonium tetrathiomolybdate into the supernatant of Ti_3_C_2_T_x_ and mixed them homogeneously by ultrasonication [94]. The mixed solution was then immersed in liquid nitrogen and subsequently annealed to prepare MoS_2_/Ti_3_C_2_T_X_ nanoscrolls. The as-prepared MoS_2_/Ti_3_C_2_T_X_ hybrid nanoscrolls exhibited an overpotential of 152 mV at a current density of 10 mA·cm^−2^ and a Tafel slope of 70 mV·dec^−1^ (Figure 32e,f). Compared with a single-layer MoS_2_ nanosheet, the exchange current density of the MoS_2_/Ti_3_C_2_T_X_ hybrid nanoscroll increased more than 25-fold [94].

The crystalline phase of TMDC nanoscrolls also affects HER performance. Wang et al. prepared metallic-phase WSe_2_ (M-WSe_2_) nanoscrolls using Li-intercalation exfoliation in combination with a spontaneously curling process. Compared with 2H-WSe_2_ nanoscrolls prepared by a thermal annealing method, the electrocatalytic performance of M-WSe_2_ nanoscrolls was greatly improved. At a current density of 10 mA·cm^−2^, the M-WSe_2_ nanoscrolls had an overpotential of 282 mV, whereas that of the 2H-WSe_2_ nanoscrolls was 401 mV (Figure 32g). The Tafel slopes of M-WSe_2_ and 2H-WSe_2_ nanoscrolls were 82.3 and 147.7 mV·dec^−1^ (Figure 32h), respectively, indicating the good electrocatalytic performance of the M-WSe_2_ nanoscrolls. In addition, the M-WSe_2_ nanoscrolls exhibited low resistance and good stability [95]. Similarly, Hwang et al. demonstrated that MoS_2_@Pt nanoscrolls with mixed 1T/2H phases exhibited excellent catalytic activity [55]. They found that a MoS_2_ nanosheet showed a high Tafel slope of 167 mV·dec^−1^. In contrast, the Tafel slope of 1T/2H-MoS_2_@Pt nanoscrolls decreased significantly to 39 mV·dec^−1^, indicating the important role of the crystalline phase in enhancing electrocatalytic performance.

### 5.4. Gas Sensor

**Figure 33:**
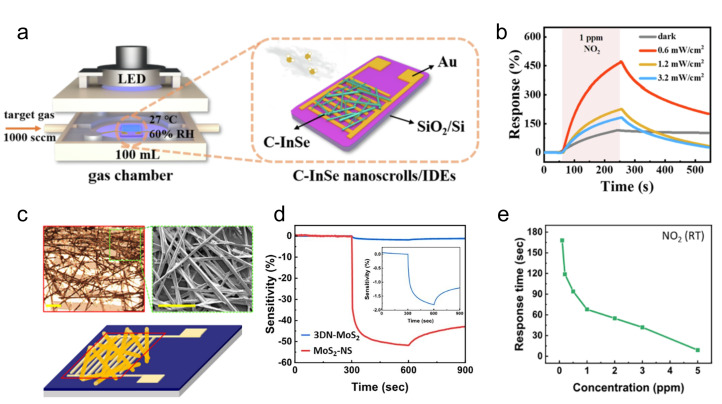
TMDC nanoscrolls for gas sensing. (**a**) Schematic of a C-InSe nanoscroll-based gas sensor. (**b**) Response curves of a C-InSe nanoscroll-based sensor exposed to 1 ppm NO_2_ under illumination from a blue LED. Reproduced with permission from Ref. [61]. Copyright 2022 Elsevier. (**c**) Optical, SEM, and schematic images of a 3DN-MoS_2_ NS-based gas sensor. Scale bar: 200 μm. (**d**) Sensitivity plots of a 3DN-MoS_2_ film and a 3DN-MoS_2_ NS-based sensors exposed to 5 ppm NO_2_. (**e**) Response time of a 3DN-MoS_2_ NS-based sensor as a function of NO_2_ concentration at room temperature (RT). Reproduced with permission from Ref. [30]. Copyright 2023 Elsevier.

Compared with 2D TMDC nanosheets, 1D TMDC nanoscrolls possess multiple inner and outer surfaces, which form double depletion regions for gas absorption and desorption, leading to an improved change in resistance. Therefore, TMDC nanoscrolls are considered to exhibit better gas-sensing performance than TMDC nanosheets. Zhang et al. prepared a gas sensor based on carbon/oxygen functional-group-modified InSe (C-InSe) nanoscrolls (Figure 33a) [61]. InSe nanosheets were exfoliated by electrochemical intercalation and ultrasonication. After scrolling the InSe nanosheets by solvent evaporation at 80 °C under vacuum, the samples were heated at 300 °C for 2 h to obtain C-InSe nanoscrolls. Benefiting from the unique loosely scrolled structure and excellent optoelectronic properties, the C-InSe nanoscrolls exhibited response and recovery times twice as fast as those of C-InSe nanosheets. Under visible light illumination, the C-InSe nanoscrolls exhibited a response intensity of 381% per ppm NO_2_ (Figure 33b), a recovery time of 200 s, a detection limit as low as 0.43 ppb, good selectivity, repeatability, and long-term stability. The excellent gas-sensing performance of C-InSe nanoscrolls can be explained as follows. First, the tubular structure of the nanoscroll increases the specific surface area and provides more active sites for gas absorption and desorption. Second, internal reflection of light within the nanoscroll cavity enhances light absorption and increases the carrier concentration. Third, the carbon/oxygen functional groups of the nanoscroll enhance the binding energy of NO_2_ through chemical interactions and simultaneously accelerate desorption. Therefore, TMDC nanoscrolls provide a new strategy for constructing gas-sensing platforms with high stability and low power consumption.

Park et al. rolled up the three-dimensionally nanostructured MoS_2_ (3DN-MoS_2_) film by dropping ethanol solution [30]. The as-prepared 3DN-MoS_2_ nanoscrolls (3DN-MoS_2_ NS) were used as sensitive channel materials for a gas sensor to detect NO_2_ (Figure 33c). Owing to the increased surface area and exposed active edges, the 3DN-MoS_2_ NS exhibited enhanced gas-sensing performance compared with the 3DN-MoS_2_ film. As an oxidizing gas, NO_2_ acts as an electron acceptor. Both the 3DN-MoS_2_ film and the 3DN-MoS_2_ NS sensors exhibited decreased resistance because of their p-type character, indicating negative sensitivity (Figure 33d). Under 5 ppm NO_2_, the sensitivities of the 3DN-MoS_2_ NS and the 3DN-MoS_2_ film were 51% and 1.8%, respectively, indicating a 28-fold enhancement in sensitivity for the nanoscroll. As the concentration of NO_2_ increased from 100 ppb to 5 ppm at room temperature, the response time of the 3DN-MoS_2_ NS decreased from 168 s to 9 s (Figure 33e), implying that a low concentration of NO_2_ leads to a decreased gas diffusion rate in the interlayer spaces of the nanoscroll and a delayed response time [30].

### 5.5. Surface-Enhanced Raman Scattering

When molecules are positioned close to the surface of gold or silver nanostructures, their Raman scattering signals are greatly enhanced. This phenomenon is known as surface-enhanced Raman scattering (SERS) [96]. Owing to their atomic thickness and high surface activity, TMDCs are often explored in the field of SERS. However, the SERS performance of monolayer MoS_2_ is limited owing to weak charge transfer, rapid charge recombination, and low conductivity [97].

**Figure 34:**
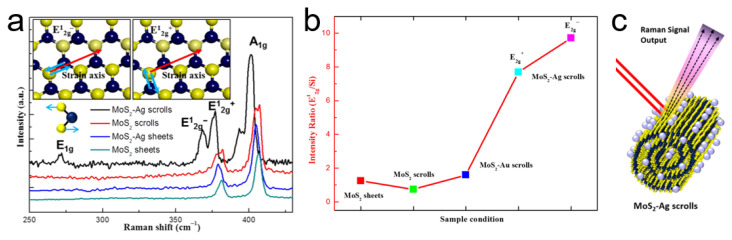
MoS_2_ nanoscroll for surface-enhanced Raman scattering. (**a**) Raman spectra of a MoS_2_ sheet, a MoS_2_ nanoscroll, and a MoS_2_-Ag nanoscroll excited by a 532 nm laser. (**b**) Raman intensity ratios of $E_{2g}^{1}$/Si, $E_{{2g}^{+}}^{1}$/Si, and $E_{{2g}^{-}}^{1}$/Si for a MoS_2_ sheet, a MoS_2_ nanoscroll, and a MoS_2_-Ag nanoscroll. (**c**) Schematic of the Raman measurement for a MoS_2_-Ag nanoscroll. Reproduced with permission from Ref. [56]. Copyright 2017 IOP.

Encapsulating Ag and Au nanoparticles (NPs) into MoS_2_ nanoscrolls enables a large SERS enhancement. When Ag and Au NPs are encapsulated into MoS_2_ nanoscrolls to form MoS_2_-Au and MoS_2_-Ag nanoscrolls, the tensile strain induced by the curved scroll structure generates a local electric field accompanying surface plasmon excitation, resulting in enhanced SERS performance [56]. As shown in Figure 34a, the $E_{2g}^{1}$ peak of the MoS_2_-Ag nanoscroll split into $E_{{2g}^{+}}^{1}$ and $E_{{2g}^{-}}^{1}$ peaks, which have also been reported in MoS_2_ nanosheets under tensile strain [98]. Thus, the appearance of the two new peaks, $E_{{2g}^{+}}^{1}$ and $E_{{2g}^{-}}^{1}$, arises from the uniaxial strain induced in the MoS_2_-Ag nanoscroll. Furthermore, an in-plane $E_{1g}$ peak was observed at 274 cm^−1^, which is Raman inactive both for MoS_2_-Ag nanosheets and MoS_2_ nanoscrolls. When a molecule is positioned close to a Ag NP, the electronic interaction between the molecule’s orbitals and the NP’s conduction band activates a new charge-transfer resonance, which couples to the vibrational state of the molecule. Consequently, the vibrational motion and electron density within the molecule are redistributed, creating a localized surface plasmon resonance and improving SERS performance [56,96]. As shown in Figure 34b, the intensity ratios of $E_{{2g}^{+}}^{1}$/Si and $E_{{2g}^{-}}^{1}$/Si for MoS_2_-Ag nanoscroll were 7.69 and 9.71, respectively, which were much higher than the intensity ratio of $E_{2g}^{1}$/Si for a MoS_2_ nanosheet (~1.2). Furthermore, the intensity ratio of $A_{1g}$/Si for the MoS_2_-Ag nanoscroll was approximately 24.3, roughly 7 times higher than that of a MoS_2_ nanosheet. The enhanced Raman peaks of the MoS_2_-Ag nanoscroll are attributed to the tensile strain arising from bending of the MoS_2_ layer caused by the encapsulated Ag NPs (Figure 34c). Moreover, the SERS enhancement factor was calculated to be as high as 1.22 × 10^5^.

### 5.6. Bragg Reflector

**Figure 35:**
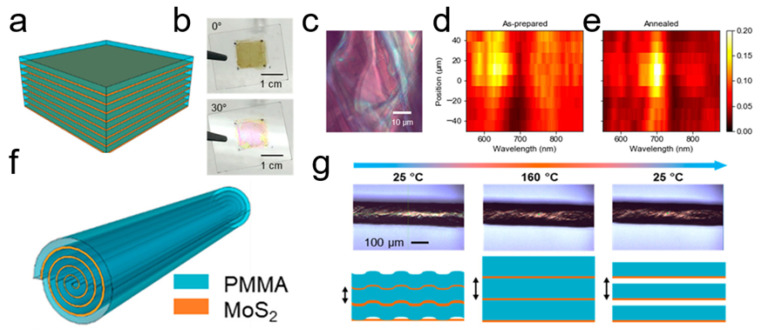
Bragg reflector of a MoS_2_/PMMA nanoscroll fiber. (**a**) Schematic of a planar MoS_2_/PMMA heterostructure. (**b**) Optical photographs of the heterostructure film tilted at 0° and 30°. (**c**) Optical microscopy image of a MoS_2_/PMMA nanoscroll fiber. (**d**,**e**) Hyperspectral images of the MoS_2_/PMMA nanoscroll fiber (**d**) before and (**e**) after annealing at 160 °C. (**f**) Schematic structure of a MoS_2_/PMMA nanoscroll fiber. (**g**) Optical micrographs and corresponding schematics of the MoS_2_/PMMA nanoscroll fiber at 25 °C and 160 °C, and after cooling back to 25 °C. Reproduced with permission from Ref. [16]. Copyright 2020 ACS.

When multiple layers with different refractive indices are periodically stacked, they form a Bragg reflector. When such a Bragg reflector is irradiated with incident light, multiple interferences are observed. Under 532 nm irradiation, the refractive indices of MoS_2_ and poly (methyl methacrylate) (PMMA) are 4.43 and 1.49, respectively. When MoS_2_ and PMMA layers are alternatively stacked to form a planar structure (Figure 35a), a Bragg reflector is constructed, which can enhance the light-coupling efficiency of 2D TMDCs [99]. The planar MoS_2_/PMMA stack appears brown under white illumination and exhibits red reflection when tilted by 30°, which arises from Bragg diffraction (Figure 35b) [16]. A monolayer MoS_2_ exhibits PL peaks at 603 and 651 nm. In contrast, the planar MoS_2_/PMMA stack exhibits a Bragg wavelength (λ_B_) at 636.7 nm. A monolayer MoS_2_ on a 226 nm-thick PMMA film was scrolled using a transverse shear method to obtain a scroll fiber with a diameter of approximately 81 µm (Figure 35c,f). The MoS_2_/PMMA scroll fiber exhibited red reflection with a λ_B_ of 629.1 nm under white illumination (Figure 35d). The λ_B_ significantly red-shifted to 697.8 nm after the MoS_2_/PMMA scroll fiber was annealed at 160 °C (Figure 35e), a temperature higher than the glass transition temperature of PMMA (100–120 °C). The redshift of λ_B_ can be explained as follows. Unlike the planar MoS_2_/PMMA stack, wrinkles inevitably formed on the PMMA layers during the transverse shear scrolling process. The wrinkles in the PMMA layers could be stretched above the glass transition temperature, and air gaps formed between the MoS_2_/PMMA layers after cooling to room temperature (Figure 35g).

### 5.7. Synapse

**Figure 36:**
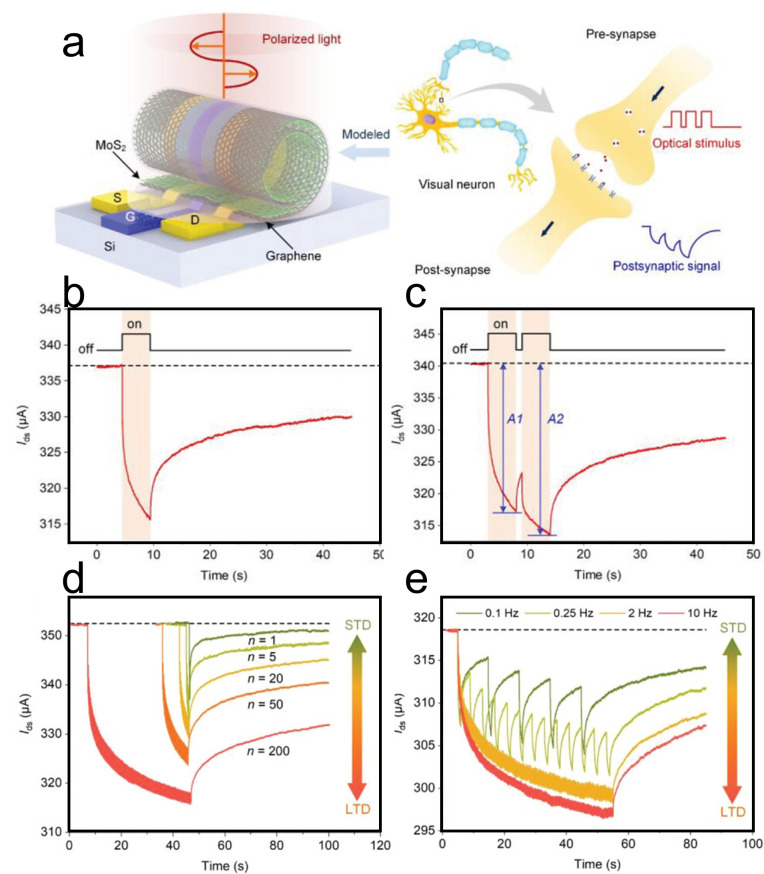
Synapse emulation using a graphene/MoS_2_ nanoscroll. (**a**) Schematic illustration of synapse emulation using a graphene/MoS_2_ nanoscroll-based FET. (**b**,**c**) Photoresponse characteristics of the graphene/MoS_2_ nanoscroll-based FET under (**b**) optical switching and (**c**) a light pulse pair with an interval of 1s illuminated by 660 nm light. (**d**,**e**) Transition from short-term depression (STD) to long-term depression (LTD) under repeated optical stimulation with different (**d**) pulse numbers and (**b**) frequencies. Reproduced with permission from Ref. [104]. Copyright 2023 Wiley.

By mimicking the function of biological neural synapses, the optoelectronic synapse plays a key role in advancing neuromorphic and brain-inspired computing [100,101,102,103], serving as the core component of artificial visual perception systems. Such a device can not only sense and process optical signals but also mimic the information perception, processing, and memory of a neuromorphic system through electrical signals. Li et al. developed a polarization-sensitive optoelectronic synapse based on a graphene/MoS_2_ heterojunction field-effect transistor (FET) with a scrolled tubular structure [104] (Figure 36a). The presence of graphene greatly enhanced the carrier mobility of the device. Owing to its broad light absorption range spanning from UV to visible spectra, MoS_2_ exhibits sustained photoconductivity, making it suitable for emulating a wide range of neural synaptic functions. The resulting graphene/MoS_2_ scroll structure, with its multiple heterojunction interfaces, further enhances light absorption and polarization sensitivity. Numerous defects exist at the graphene/MoS_2_ heterojunction interfaces and on the MoS_2_ surface, which can trap photogenerated carriers for a finite period, leading to a long recovery time (Figure 36b). Consequently, the relaxation of photocurrent can be used to emulate the postsynaptic current (PSC) in biological synapses. When the FET device based on the graphene/MoS_2_ heterojunction scroll was excited again by a light pulse while some photogenerated carriers remained trapped by the defects, a longer recovery time was observed (Figure 36c), which can be used to emulate synaptic plasticity in a biological system. Using two consecutive light pulses under 660 nm illumination with a pulse width of 5 s and an interval of 1 s, the amplitude of the second photocurrent (A2) was higher than that of the first photocurrent (A1) (Figure 36c). The human brain exhibits two forms of synaptic plasticity, which are considered the fundamental mechanisms underlying learning and memory. Information encoded by short-term potentiation (STP) is readily forgotten because STP is associated with transient neural responses. In contrast, external information can be stored for extended periods through long-term potentiation (LTP) via repeated rehearsal. Under repetitive light pulses at various frequencies, the graphene/MoS_2_ heterojunction scroll can also emulate short-term depression (STD), long-term depression (LTD), and the STD-LTD transition (Figure 36d,e).

This section presents the broad application prospects of TMDC nanoscrolls in photodetectors, miniaturized memory, electrocatalytic hydrogen evolution reaction, gas sensing, surface-enhanced Raman scattering, Bragg reflectors, and synaptic devices. The unique performance of TMDC nanoscrolls in these applications stems from their distinctive geometric structure, strain effects, and interlayer coupling.

The rolled structure of TMDC nanoscrolls enhances light–matter interaction, and scrolling induces strain-tunable bandgaps, greatly improving the photodetection responsivity (PDR) and detectivity. For example, QD/WS_2_ nanoscrolls achieve a responsivity of 1.67 × 10^4^ A/W, substantially higher than that of planar QD/WS_2_ nanosheets. The PDR of MoS_2_ nanoscrolls is two orders of magnitude higher than that of MoS_2_ nanosheets. Moreover, WSe_2_ nanoscrolls exhibit strong polarization-sensitive properties with an anisotropy ratio of 1.5–2.0. Self-powered polarization imaging has been realized using a 1D-WSe_2_ nanoscroll/2D-WSe_2_ nanosheet homojunction, a capability unattainable with WSe_2_ nanosheets alone. The edges of TMDC nanoscrolls provide abundant active sites, and the reduced interlayer barrier facilitates electron transport, lowering the hydrogen evolution overpotential of MoS_2_ nanoscrolls from −343 mV to −153 mV. Notably, MoS_2_ nanoscrolls with mixed 1T/2H phases or WS_2_/MoS_2_ heterojunction nanoscrolls further enhance catalytic efficiency. The inner and outer surfaces of a nanoscroll form a double depletion layer, and the carrier concentration increases under illumination, enabling a C-InSe nanoscroll to achieve an ultra-low detection limit of 0.43 ppb for NO_2_. The defect trapping effect in curled graphene/MoS_2_ heterojunctions accurately mimics short-term and long-term plasticity, providing new hardware support for neuromorphic computing.

Despite their excellent performance, current research remains largely at the proof-of-concept stage, with few studies successfully integrating nanoscrolls into practical systems. The core obstacle lies in the large-scale preparation of TMDC nanoscrolls with high yield, low cost, and ease of operation.

## 6. Conclusions

**Figure 37:**
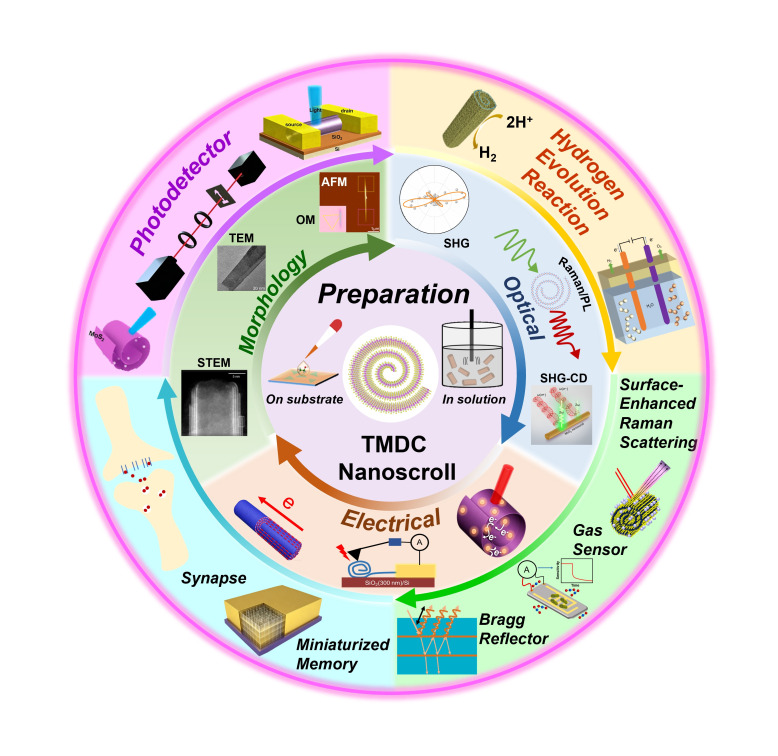
Summary of the preparation, properties and applications of TMDC nanoscrolls.

Over the past decade, transition metal dichalcogenide nanoscrolls (TMDC nanoscrolls) have attracted much attention and become a focus of intense research, owing to their fascinating and distinctive 1D architecture. By transforming flat 2D nanosheets into spiraled 1D structures with a hollow core, TMDC nanoscrolls not only inherit the intrinsic electronic, optical, and catalytic properties of the parent 2D materials, but also possess geometric advantages of 1D rolled structure. Compared with flat TMDC nanosheets and conventional nanotubes, TMDC nanoscrolls exhibit unique features rarely found together in a single nanomaterial. First, the spiral morphology yields a massively enhanced accessible surface area. The outer wall, the inner core wall, and the interlayer spacings between successive turns all become available for interaction. Second, exceptionally high density of catalytically active edge sites is exposed along the entire scroll, which is superior to the hydrogen evolution reaction (HER). Third, the curvature of scroll wall can be precisely tuned by changing interlayer spacing, enabling continuous modulation of the bandgap, excitonic behavior, and catalytic adsorption energies. Finally, the hollow core and the open interlayer spacings form continuous nanochannels along the scroll axis, facilitating rapid longitudinal mass transport of ions, molecules, and gases, which can overcome the diffusion bottlenecks of stacked 2D materials. This review systematically discusses the preparation, properties, and applications of TMDC nanoscrolls. Regarding preparation, we present a critical cross-method comparison with quantitative assessments of scalability, cost, and yield. We also provide a systematic investigation of the properties of TMDC nanoscrolls, including SHG, CD, ultra-low-frequency Raman, magnetoresistance, and others. Quantitative benchmarking tables are summarized to expand the application landscape to include memory, SERS, Bragg reflectors, and synapses. These features collectively distinguish our review from previous efforts. A graphical roadmap summarizing the preparation, properties, and applications of TMDC nanoscrolls is shown in Figure 37.

Despite these compelling attributes, the path to practical application of TMDC nanoscrolls is obstructed by some challenges. First, the scalable and controlled synthesis of TMDC nanoscrolls suffers from low yield and poor uniformity. Preparing gram-scale quantities of TMDC nanoscrolls with precisely defined dimensions and high purity is difficult to achieve. Key structural parameters of TMDC nanoscrolls, such as diameter, length, number of layers, pitch, and chirality, are hard to control efficiently. Second, nondestructive characterization of the complex three-dimensional structure of TMDC nanoscrolls is highly dependent on sophisticated techniques like aberration-corrected transmission electron microscopy. Third, long-term structural and operational stability is still unresolved under high applied potential in the oxidative environment during electrochemical cycling. Finally, integrating individual scrolls into large-area arrays for functional devices remains technically challenging.

Addressing these limitations will require coordinated advances across synthesis, characterization, and materials engineering. A deeper fundamental understanding must accompany these synthetic efforts. Advanced in situ and operando characterization tools (liquid-cell electron microscopy, electrochemical atomic force microscopy, operando Raman and X-ray absorption spectroscopy) will be essential to observe structural evolution, strain dynamics, and chemical transformations under realistic operating conditions. Multi-scale modeling, linking density functional theory (DFT) with molecular dynamics and continuum mechanics, can provide predictive relationships between scroll geometry and performance, guiding the rational design of optimized structures. Stability can be enhanced through protective coatings (ultrathin carbon, conductive polymers, atomic-layer-deposited oxides) that shield scrolls without blocking active sites, and through integration into robust three-dimensional frameworks such as graphene foams or carbon nanotube networks. Finally, device integration must advance from isolated demonstrations to engineered systems.

Transition metal dichalcogenide nanoscrolls stand at an exciting crossroads. Their unique combination of high accessible surface area, abundant edge sites, tunable strain and interlayer spacing, open mass-transport channels, and structural resilience offers a platform that surpasses both their 2D precursors and conventional 1D counterparts. Yet the journey from these intrinsic advantages to real-world impact is contingent upon overcoming the synthesis, stability, and integration hurdles that currently define the field. The coming decade will likely witness a convergence of scalable manufacturing, advanced in situ characterization, and predictive modeling, transforming TMDC nanoscrolls from a collection of proof-of-concept demonstrations into enabling components for sustainable energy systems, smart sensors, and next-generation quantum devices. Their evolution will serve as a testament to the enduring value of dimensional engineering in nanomaterials research.

## Figures and Tables

**Table 1:** Methods for Preparing TMDC Nanoscroll.

Method		Advantages	Challenges	Length	Driving Force	Ref.
On substrate	Organic solvent evaporation	Large size, high yield, and short time	Solvent residue, loose structure, and degraded optoelectronic performance	a few hundred micrometers	Marangoni flow	[17,18,19,30,31,32,33,35,36,37,38]
	Alkaline droplet-assisted	High yield and suitable for thick nanosheets	Substrate etching and solvent residue	a few hundred micrometers	Reduced adhesion	[12,40,41,42]
	Water droplet dragging	Solvent free and tightly packed structure	Not suitable for moisture or temperature sensitive materials	several tens of micrometers	Liquid flow	[39]
	Spin-coating	Large scale, compact structure, and environment friendly	Applicable to monolayer nanosheet	a few hundred micrometers	Liquid flow	[20]
	Plasma-assisted	Simple process and high yield	Small size and structural damage	less than 1 μm	Lattice distortion	[22,23]
	Quenching-induced	Simple process	Complex process, low yield, and incomplete curling	~8 μm	Thermal expansion coefficient difference induced strain	[44]
In solution	Vortex fluidic device (VFD)	High yield and easy operation	Solvent residue	~10 μm	Strong shear force	[45]
	Sonication	Simple, low cost, and scalable	Small size	~650 nm	Impact stress	[48]
	Supercritical fluid	Simple and short processing time	Small size and solvent residue	0.2–3 μm	Surface energy miniaturization	[51,52]
	LCA self-assembly	High yield and easy operation	Small size and solvent residue	0.5–2 μm	Local strain	[53,54,55,56]
	Pulsed laser ablation (PLAL)	Rapid and low cost	Low yield and oxide byproducts	~500 nm	Overcoming dynamic hydrogen bonds	[57]
	Magnetic stirring	Facile and scalable	Solvent residue	0.5–10 μm	Edge localized particles	[60]

**Table 2:** Preparation comparison of TMDC Nanoscroll.

Nanoscroll Type	Preparation Method	Temperature	Dimensions	Ref.
MoS_2_ (also WS_2_)	Organic solvent evaporation	Room temperature (RT)	Diameter: 314 nm; Length: a few hundred micrometers	[17,18,30,31,32,33]
	Dragging water droplet	100 °C	Diameter: 245.1 nm; Length: several tens of micrometers	[39]
	Spin coating PEG droplet	RT	Height: 36 nm; Length: a few hundred micrometers	[20]
	Plasma bombardment (S removal)	150 °C	Height: 14.6 nm; Length: ~500 nm	[22,23]
	Rapid quenching (300 °C/min)	Not applicable	Height: 14 nm; Length: 8 μm	[44]
	Vortex fluidic device (VFD)	RT	Length: 10 μm	[45]
WS_2_	Ultrasonication	RT	Height: 5–10 nm; Length: 650 nm	[48]
	Supercritical fluid	400 °C	Diameter: 50–150 nm; Length: 0.2–3 μm	[51,52]
	LCA self-assembly	RT	Diameter: 20 nm; Length: 0.5–2 μm	[53,54,55,56]
WSe_2_	Pulsed laser ablation (PLA)	70 °C	Length: ~500 nm	[57]
Janus MoSSe	H_2_ plasma (top Se replaced by S) + spin-coating PMMA/chloroform	RT	Height: 8–61 nm; Length: 0.18–2.3 μm	[36]
InSe	Solvent assisted self-assembly	80 °C	Diameter: 10.5 nm; Length: 90 μm	[61]
MoS_2_/WS_2_	Alkaline solution etching	RT	Diameter: ~100 nm; Length: a few hundred micrometers	[12]
SnS_2_/WSe_2_	Alkaline solution etching	RT	Length: 3–7 μm	[40]

**Table 3:** Performance of TMDC Nanoscroll Photodetectors Wrapped with Functional Materials.

TMDC Nanoscrolls	Functional Materials	Performance Improvement
Organic material-wrapped	R6G (rhodamine)	At 405 nm: responsivity (R), EQE, and detectivity (D*) are four orders of magnitude higher than monolayer TMDC
Inorganic material-wrapped	CQDs (carbon quantum dots)	Under 300 nm: photocurrent increased by 20.7 times; R increased by 830 times (up to 1793 A/W); specific detectivity (D*) increased by 268 times; EQE increased by 830 times; under 400 nm: photocurrent increased by 10.7 times
	BaTiO_3_	Photoresponsivity (73.9 A/W) significantly higher than pure MoS_2_ nanoscroll (1.1 A/W) and 2D MoS_2_ nanosheet (1.5 A/W)
	PbI_2_	PDR improved by two orders of magnitude vs. pure MoS_2_ nanosheets and nanoscrolls. Under 405 nm: PDR is 91 times that of MoS_2_ nanoscroll
	Ag^+^	PDR increased up to 530 times compared with monolayer TMDC nanosheet (under 633 nm laser)
	WS_2_/MoS_2_ heterojunction	PDR increased by 15 times; shorter response and recovery time
	WSe_2_ homojunction	Excellent performance under zero bias: on/off ratio = 1.5 × 10^3^; detectivity = 3.24 × 10^9^ Jones

## Data Availability

No new data were created or analyzed in this study. Data sharing is not applicable to this article.
